# A Fast and Convenient Synthesis of New Water-Soluble, Polyanionic Dendrimers

**DOI:** 10.3390/molecules26164754

**Published:** 2021-08-06

**Authors:** Grzegorz M. Salamończyk

**Affiliations:** Centre of Molecular and Macromolecular Studies, The Polish Academy of Sciences, Sienkiewicza 112, 90-363 Łódź, Poland; gmsalamo@cbmm.lodz.pl; Tel.: +48-42-684-7113; Fax: +48-42-684-7126

**Keywords:** dendrimer, polyanion, 1,3,5-benzenetricarboxylic acid, polyester, phosphorus, total synthesis, chemoselectivity

## Abstract

Reasonably simple, efficient, and possessing aspects of generality, the methodology for the synthesis of new, water-soluble, dendrimeric polyesters with great potential applications as antiviral drugs in their own right is described. The essential aspect of the presented approach is a quite unique, immediate access to the polyanionic material at each generation during divergent synthesis. Six target polyanionic dendrimers (generations 1, 2, and 3) have been synthesized. The key monomers applied in this project were 1,3,5-benzenetricarboxylic acid derivatives, which also worked as direct precursors of the charged dendrimer surface.

## 1. Introduction

Dendrimers are three-dimensional, highly branched, synthetic polymers that possess a well-defined structure. Their graphical representation resembles tree-like units emanating from a common spot. Naturally, dendrimers have been extensively studied for a variety of biomedical applications involving both therapy and diagnosis. However, this research seems to be still at an early stage [1,2,3]. Normally, antiviral and other drug research concentrates on relatively low molecular weight structures. On the contrary, dendrimers with their structural precision and with a large number of functional groups on the surface may serve as antiviral drugs in their own right [4,5,6]. Different types and generations of dendrimers substituted with different charged groups have been explored in the prevention and treatment of HIV. These macromolecular drug candidates can be administered as topical microbicides [7,8,9]. Topical microbicides interfere with the virus at the early stage of virus infection sequence, which is adsorption and fusion of the virus to the cell. The common feature of all such microbicide compounds is the presence of polyanions, which have the capability to bind to the (e.g., the so-called gp120 protein or spike protein (in the case of SARS-CoV-2)) outer protein located on the viral surface, thus inhibiting the entire viral infection process [10]. There are numerous examples of this type of compounds that can be found in the literature [11,12,13,14]. Thanks to their mode of action, polyanionic dendrimers can be expected to be effective against a broad spectrum of pathogenic viruses. In contrast to polycationic compounds, polyanionic dendrimers usually possess much lower cytotoxicity [15,16]. Definitely, the most successful polyanionic dendrimer used for antiviral purposes is SPL7013 (also known as VivaGel^®^ or VIRALEZE™—Starpharma Ltd. A company developing dendrimer products for pharmaceutical applications, Australia), a sulfonated (32 terminal sulfonate groups) polylysine dendrimer (molec. weight 16.6 kD) [17]. VivaGel^®^ was originally developed as a topical microbicide for the avoidance of sexually transmitted infections (STIs) such as HIV, HSV-2 (genital herpes), and human papillomavirus (HPV). There is an enormous global need for a vaginal microbicide offering protection of this nature. It has also been licensed as an antiviral condom coating. This compound went effectively through all the stages of clinical trials [18]. Except STIs causing viruses, the SPL7013 dendrimer possesses significant activity against other pathogenic viruses such as adenovirus, HBV (hepatitis B), RSV (respiratory syncytial virus) or Zika virus. Moreover, in April 2020, it was declared that the same dendrimer SPL 7013 showed significant antiviral activity against the coronavirus (SARS-CoV-2) that causes COVID-19 (coronavirus disease) [19]. Later on, in September 2020, Starpharma announced that SPL 7013 when applied at clinically relevant concentrations as a nasal spray (VIRALEZE™), had potent virucidal activity, inactivating more than 99.9% of highly infectious SARS-CoV-2.9 [20]. Therefore, VIRALEZE™ can be complementary to vaccines and other preventive measures.

Consequently, the chemical synthesis of potential antiviral compounds, including macromolecular material, such as water-soluble, charged dendrimers, possessing various types of tunable scaffolds and surfaces, is extremely important and very much justified.

## 2. Results and Discussion

Several years ago, we developed a method for the synthesis of dendrimeric polyphosphates and their analogs [21,22,23,24,25]. Later on, also from our laboratory, an effective syntheses of new polyester dendrimers based on a trimesic acid framework derivative have been disclosed [26,27]. In this research paper, a general and undemanding approach to the synthesis of carboxylate-terminated (polyanionic) dendrimers is reported. The key feature of this approach is the unprecedented formation and straightforward access to a polyanionic dendrimer at each generation during the divergent synthesis. Therefore, there is no need for often complicated, post-synthetic surface modification, and at least one difficult synthetic step is saved. Recently, we have found that medium-size (4.2–4.7 kD) carboxylate-terminated polyanionic dendrimers [28] displayed exceedingly strong antiviral activity against both strains of HIV, way below their cytotoxicity versus MT4 cells [29]. Therefore, for a powerful antiviral activity, the high generation structures are not necessarily essential.

### 2.1. Synthesis of Monomers

**Scheme 1:**
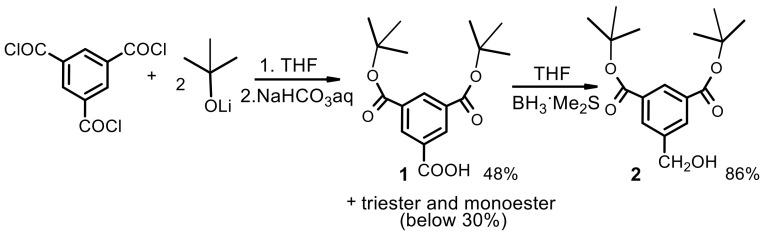
Synthesis of the monomers **1** and **2**.

The presented synthetic work commences from the preparation of both interior and surface unit monomers. The careful reaction of commercially available 1,3,5-benzenetricarbonyl trichloride with 2.0 equiv. of lithium *tert*-butoxide, followed by a mild basic hydrolysis of the remaining acid chloride, produced chemoselectively the corresponding 1,3,5-benzenetricarboxylic acid di-*tert*-butyl ester or di-*tert*-butyl trimesoate (**1**) (48%).

(Accompanied with the corresponding triester (11%) and monoester (17%), acc. to NMR) in 48% isolated yield. (Scheme 1) The synthesis (very low yield—22% in a two-step procedure) of diester **1** has been recently reported [30]. Another highly chemoselective reaction was a reduction (rt, 24 h) of one carboxyl group in diester **1** using the borane–dimethyl sulfide complex, which provided the key reagent—di-*tert*-butyl 5-hydroxymethylbenzene-1,3-dicarboxylate (**2**) in a high isolated yield (86% after crystallization from CH_2_Cl_2_–cyclohexane). This compound (**2**) represents an AB_2_-type monomer. The A group (hydroxyl) is active and the B groups (carboxyl) are protected such that the A group reacts solely with the B (active) groups in the prior generation of the dendrimer. Deprotection is necessary to activate the B groups for the consequent reaction. This deprotection may not be quantitative and may also include undesired reactions, causing imperfections in the dendrimer skeleton. Therefore, the protective groups applied in this work were selected very cautiously. It will be demonstrated that deprotection reactions (cleavage of *tert*-butyl esters in the presence of benzoate-type esters) were complete and did not cause any defects in the expected structures.

### 2.2. Synthesis of Dendrimers

To express the usefulness of the presented synthetic methodology, the author split this project into three subsections.

**Scheme 2:**
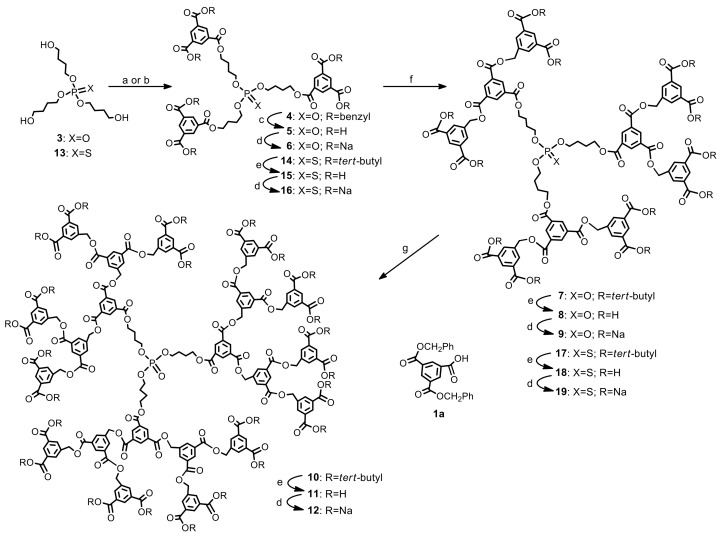
Reagents and conditions: (a) for **3** as the substrate: acid **1a**, EDC, DMAP, DCM, rt; (b) for **13** as the substrate: acid **1**, EDC, DMAP, DCM, rt; (c) H_2_, Pd/C, MeOH; (d) NaHCO_3_ aq.; (e) CF_3_COOH, DCM, rt; (f) alcohol **2**, EDC, DMAP, DCM, THF, rt; (g) for dodecaacid **8** as the substrate: alcohol **2**, EDC, DMAP, DCM, DMF, rt.

Firstly, the core compound, tris(4-hydroxybutyl) phosphate [28] (**3**) was allowed to react with an excess of dibenzyl 1,3,5-benzenetricarboxylate [28], (**1a**) in the presence of the water-soluble carbodiimide, 1-ethyl-3-(3-dimethylaminopropyl)carbodiimide (EDC) and 4-dimethylaminopyridine (DMAP) in anhydrous dichloromethane to furnish the first generation (G1) dendrimer **4** in 92% isolated yield. (Scheme 2) The cleavage of the terminal benzyl esters in **4** using catalytic hydrogenolysis proceeded smoothly and quantitatively as evidenced by the ^1^H NMR to provide pure acid **5** (no trace of protons corresponding to benzyl groups in **5** was detected) (96%), which can be effortlessly transformed into the corresponding hexaanion **6** using, e.g., aqueous sodium bicarbonate. Then, hexacarboxylic acid **5** reacted readily with an excess of alcohol **2** using EDC and DMAP as condensation reagents (in CH_2_Cl_2_-THF 1:1 solution) to afford the second generation (G2) dendrimer **7** in 88% isolated yield.

**Figure 1:**
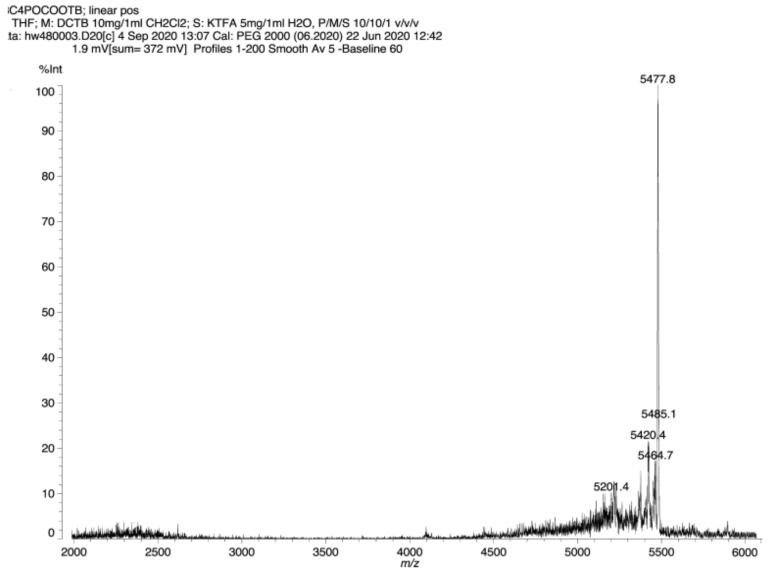
The MALDI TOF MS spectrum of dendrimer **10**.

Next, polyester **7** was stirred in a trifluoroacetic acid-dichloromethane 3:2 mixture at room temperature. The reaction was closely monitored by the ^1^H NMR. After 4 h, neither the trace of protons related to tert-butyl residues nor any damage (cleavage of an internal ester bond) to the dendrimer skeleton was detected. The expected polyacid **8** was obtained in 90% isolated yield (all of the polyacids (**5**, **8**, **11**, **15**, **18**, **22**) reported here were purified by their precipitation from a THF solution with acetone). At this stage, another polyanionic (dodecaanion) dendrimer **9** (sodium salt of **8**) was in hand. Repetition of the condensation of acid **8**, this time, with alcohol **2** provided the third generation polyester **10** with a 76% isolated yield. It should be noted that the condensation of polyacid **8** with alcohol **2** requires the DCM-DMF mixture as the solvent due to its (acid **8**) solubility issues. The structures, monodispersity, and high purity of all the dendrimeric products were confirmed by the NMR and MALDI TOF mass spectrometry. For instance, Figure 1 shows the MALDI TOF mass spectrum of third generation dendrimer **10**. The signals at 5464.7 and 5477.8 are attributed to the molecular ion (M + Na) and (M + K), respectively.

The minor peak (5420.4), which is 57 atom mass units in mass lower than the major one (M + K), presumably arose due to the insignificant fragmentation of one (out of 24) *tert*-butyl cation. The subsequent cleavage (TFA—DCM) of all *tert*-butyl esters (91%) led to the formation of third generation dendrimer **11** with 24 free carboxyl groups, which was further converted into the corresponding polyanion **12** by the reaction with aqueous sodium bicarbonate. It is worth noting that the terminal bulky *tert*-butyl groups do not cause any adverse congestion effects at the surface, and the third generation dendrimer was formed smoothly.

Secondly, when catalytic hydrogenolysis could not be used during the synthesis (such as the presence of nucleophilic sulfur in the substrate), an obvious solution was the replacement of dibenzyl ester **1a** with di-*tert*-butyl ester **1** (Scheme 2). Therefore, the reaction of another core triol, tris(4-hydroxybutyl) thiophosphate **13** [21,28] with an excess of di-*tert*-butyl trimesoate (**1**), again in the presence of the EDC and DMAP provided the first generation dendrimer **14** in 90% isolated yield. Similarly, the removal of the terminal *tert*-butyl ester groups from **14** occurred clearly in the TFA-DCM 3:2 mixture at room temperature. No trace of any desulfuration product was detected by means of the ^31^P NMR. The hexacarboxylic acid **15** as well as its sodium salt **16** were obtained in high yields. The reaction of the acid **15** with an excess of the benzyl alcohol **2** (EDC, DMAP, DCM-THF 2:1) furnished another second generation polyester **17** in 84% isolated yield. Dendrimer **17** was an evident precursor of the polyanionic dendrimer **19**, which was obtained by the use of *tert*-butyl ester cleavage in **17** (via polyacid **18**) using the reagents and conditions mentioned above.

**Scheme 3:**
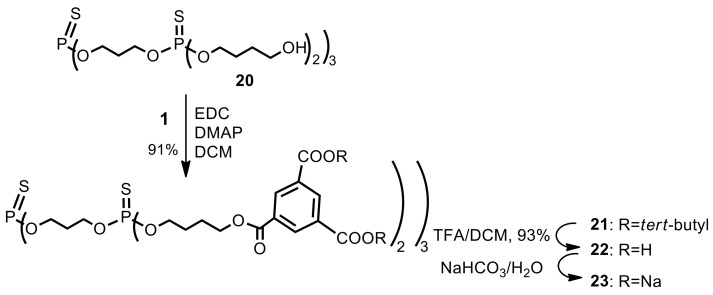
Synthesis of the second generation polyanionic dendrimer **23** and its precursors—dodecacarboxylic acid **22** and polyester **21**.

At last, di-*tert*-butyl trimesoate (**1**) can also be applied as a monomer for the modification of the surface of practically any small and medium-size dendrimer, terminated with nucleophilic functions such as: Hydroxyl, amino, thio, etc. Obviously, this conversion will eventually lead also to the polyanionic compound. A first generation hydroxyl-terminated, thiophosphate dendrimer **20** was synthesized using the amidophosphite approach [31,32,33], as described previously [21,22,28]. Therefore, the hexa-hydroxyl compound **20** possessing both four phosphorus and sulfur atoms was swiftly converted (via fully protected polyester **21**) into a second generation polyanionic dendrimer precursor–dodecaacid **22** using the two-step condensation and deprotection procedure, as described above. In the end, polyacid **22** was naturally transformed into the corresponding dodecaanion **23** using diluted aqueous sodium bicarbonate (Scheme 3).

**Figure 2:**
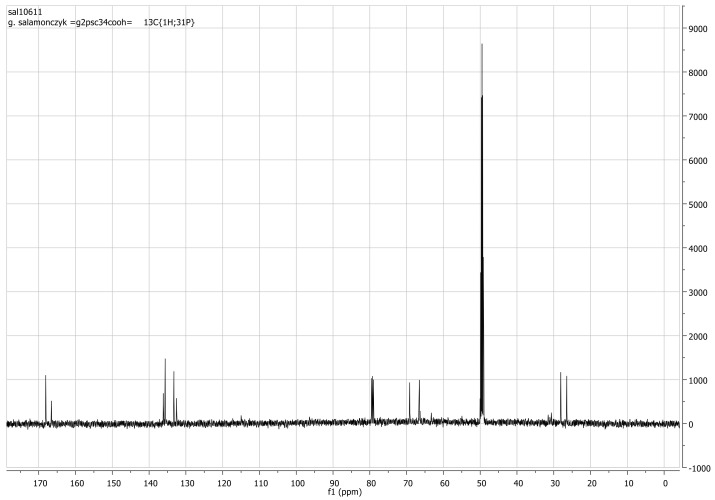
^13^C NMR spectrum of polyanionic dendrimer 22.

Dendrimers **21**–**23** are the examples of “mixed” or layered dendrimers, which have branching points both at phosphorus and at the carbon. In consequence, their ^1^H and ^13^C NMR spectra are remarkably conclusive, when compared with spectra of typical dendrimers. This is due to the diversity of the signals corresponding to nuclei absorbing in distinct areas. For instance, Figure 2 shows the ^13^C NMR (CD_3_OD-CDCl_3_ 3:1) spectrum of the polyacid **22**. Signals at 26.4 and 28.1 ppm are attributed to 12 “inner” carbon atoms from four-carbon chains, whereas the signal (multiplet, coupling with two phosphorus atoms) at 30.6–30.7 (very minor) is attributed to the three “middle” carbons of the three-carbon chains. Carbon atoms, which are in alpha position to the ester moieties, resonate at 63.4 (m, 6C, three-carbon chain), 66.5 (6C, four-carbon chain), and 69.1 (doublet, 6C four-carbon chain) ppm, respectively. The aromatic range of signals looks quite clear. There are 36 carbons distributed as follows: 6 CH, 12 CH, 12 C *ipso*, 6 C *ipso*. Finally, there are six ester carbonyls (166.6 ppm) and twelve carboxylic acid carbonyls (167.9 ppm).

The synthesized polyanionic dendrimers (**6**-G1, **9**-G2, **12**-G3, **16**-G1, **19**-G2, **23**-G2) were water-soluble non-hygroscopic white solids (powders). All of the synthesized polyanionic dendrimers possessed a decent water-solubility (at least 50 mg/1 mL). They are chemically stable in the aqueous solutions, at least, at the pH range from 5 to 9. However, as expected, for the macromolecular compounds, they were not stable enough for the melting point measurements. The synthesized dendrimers, as polyesters, have the biodegradability potential, which is critical for biomedical applications. These compounds will be tested against commonly known pathogenic viruses. The author also hopes that they will be tested against the SARS-2 coronavirus.

## 3. Materials and Methods

### 3.1. General Procedures

The melting points reported here are uncorrected and were determined using the Mel-Temp^®^ Digital Melting Point Apparatus. The NMR spectra (^1^H, ^13^C, ^31^P) were recorded on Bruker Avance AV-200, AV-500 or AV-600 spectrometers (Bruker Corporation, Billerica, MA, USA) (200, 500 or 600 MHz, respectively). Superscripts in the NMR spectra description refer to the dendrimer generation number. ^13^C NMR spectra were assisted with DEPT 90 and DEPT 135 experiments. High resolution mass spectra were recorded on the SYNAPT G2-Si (Waters Corporation, Milford, MA, USA) spectrometer and the matrix assisted laser desorption ionization time of flight mass spectra (MALDI TOF MS) were run on the MALDI AXIMA Performance ToF/ToF (Shimadzu Corporation, Kyoto, Kyoto, Japan) mass spectrometer using 2,5-dihydroxybenzoic acid as the matrix. FT-IR spectra were measured on the ATI (American Thermal Instruments, Dayton, OH, USA) Mattson Infinity 60 AR spectrophotometer. Microanalyses were performed on EuroVector 3018 and/or on Vario (Elementar Analysensysteme GmbH, Hanau, Germany) analyzers. Chemical reagents were purchased from Sigma-Aldrich and were used without any further purification. Thin layer chromatography was carried out on Merck silica gel 60F_254_ aluminum sheets using UV light (254 nm) or phosphomolybdic acid (5% solution in ethanol) for the spots visualization. Preparative flash chromatography was performed on silica gel columns (Merck, Kieselgel 230-400 mesh). The terms: “Short column” or “short pad (plug) of silica gel” used throughout this section refer to the column of silica gel with a length of 25 mm and diameter of 30 mm. Solvents were obtained from commercial sources (usually from POCh S.A., Poland) and distilled or dried according to the standard methods. The NMR and mass spectra for the synthesized compounds are shown in Supplementary Materials.

Please note, the NMR spectra of sodium salts: **6**, **9**, **12**, **16**, **19**, **23** were virtually identical with the spectra of their corresponding precursors—acids: **5**, **8**, **11**, **15**, **18**, **22**. Therefore, they are not reported.

### 3.2. Synthetic Procedures and Analyses for the Monomers 1 and 2

*Di-tert-butyl 1,3,5-benzenetricarboxylate* (**1**). To a solution of 1,3,5-benzenetricarbonyl trichloride (2.65 g, 10 mmol) in dry THF (60 mL) at −20 °C, was slowly (during about 1 h (syringe pump useful) added a 1.0 M solution of lithium *tert*-butoxide (20 mL, 20 mmol, 2 equiv.) in dry THF, and the resulting mixture was stirred for an additional 1 h, at −20 °C, then for the next 1 h at room temperature. Afterwards, a 2 M aqueous solution (15 mL) of sodium bicarbonate was added and the mixture was stirred for 30 min at room temperature. Then, the THF was removed in a vacuo and the H_2_O–MeOH mixture (2:1, 30 mL) was added to the residue and its pH was adjusted to ~10 (with 1 M NaOH). The resulting mixture was extracted with hexane (2 × 20 mL) and the hexane solutions were concentrated and the residue (tri-*tert*-butyl 1,3,5-benzenetricarboxylate 415 mg, 11%) was analyzed by the ^1^H NMR (200 MHz, CDCl_3_) δ = 1.52 (s, 27H), 8.54 (s, 3H) ppm.

Next, the organic volatiles were removed *in vacuo* once again and water (50 mL) was added to the residue and its pH was set to ~8.5 (with 0.1 M HCl). The mixture was extracted with EtOAc (4 × 30 mL). The combined EtOAc solution was concentrated. The residual material was then purified by flash chromatography using CH_2_Cl_2_-acetone (gradually increasing polarity from 30:1 to 20:1) as an eluent. Yield of **1** (white solid) 1.55 g (48%). *R_f_* 0.15 (CH_2_Cl_2_-acetone 20:1). Crystallization from the CH_2_Cl_2_-cyclohexane mixture afforded the crystalline title compound **1**; mp 202–204 °C (decomposed); IR ν max 2966, 1737, 1482, 1273, 1112, 761 cm^−1^; NMR: δ_H_ (600 MHz, CDCl3) 1.66 (s, 18H), 8.83 [t, ^3^*J*(H,H) = 1.7 Hz, 1H], 8.86 [d, ^3^*J*(H,H) = 1.7 Hz, 2H] ppm; δ_C_ (50 MHz, CDCl_3_) 28.11 {6C, [(CH_3_)_3_C)]}, 82.32 {2C, [(CH_3_)_3_C)]}, 129.9 (Ar), 132.7 [2C, (Ar)], 134.6 [2C (*ipso* Ar)], 135.2 (*ipso* Ar), 164.1 [2C, (C=O)], 170.7 (C=O) ppm; HRMS (APCI): M − H^+^, found 321.1338. C_17_H_28_O_6_ requires 321.1338.

The remaining aqueous solution was acidified to the pH ~ 3.0 with 6 M HCl, yielding a white dispersion. The organic material was then extracted with EtOAc (2 × 15 mL). The solvent was finally removed to give *tert*-butyl 1.3.5-benzenetricarboxylate as a white solid (450 mg, 17%). ^1^H NMR (500 MHz, CD_3_OD:CDCl_3_ 1:1) δ = 1.56 (s, 9H), 8.80 [d, ^4^*J*(H,H) = 1.6 Hz, 2H], 8.82 [t, ^4^*J*(H,H) = 1.6 Hz, 1H] ppm.

*Di-tert-butyl 5-hydroxymethylbenzene-1,3-dicarboxylate* (**2**). Di-*tert*-butyl 1,3,5-benzenetricarboxylate (**1**) (1.29 g, 4.0 mmol) was dissolved in dry THF (8 mL). The borane-methyl sulfide complex (4.0 mL of 2 M solution in toluene, 8.0 mmol) was added slowly, accompanied by effervescence. After 20 h of stirring at room temperature, methanol (15 mL) was added. The solution was stirred at room temperature for 30 min and concentrated under reduced pressure. The resulting white solid was dissolved in ethyl acetate (30 mL) and washed with water (25 mL) and saturated NaHCO_3_ (20 mL). The solvent was removed under reduced pressure, and the crude product was purified by a short column silica chromatography (CH_2_Cl_2_-acetone 50:1) to give **2** as a white solid (1.09 g, 88%). *R_f_* 0.29 (CH_2_Cl_2_-acetone 35:1). Crystallization from the CH_2_Cl_2_-cyclohexane mixture afforded the crystalline compound: Mp 144–145 °C; ^1^H NMR (200 MHz, CDCl_3_) δ = 1.56 (s, 9H), 4.74 (s, 2H), 8.08 [d, ^4^*J*(H,H) = 1.6 Hz, 2H], 8.42 [t, ^4^*J*(H,H) = 1.6 Hz, 1H] ppm; δ_C_ (50 MHz, CDCl_3_) 28.63 {6C, [(CH_3_)_3_C)]}, 64.59 (CH_2_OH), 82.20 {2C, [(CH_3_)_3_C)]}, 129.9 (Ar), 132.0 [2C, (Ar)], 132.9 [2C (*ipso* Ar)], 142.3 (*ipso* Ar), 165.7 [2C, (C=O)]; HRMS (APCI): M − H^+^, found 307.1541. C_17_H_28_O_6_ requires 307.1545.

### 3.3. Synthetic Procedures and Analyses for Dendrimers: **4**, **5**, **6**, **7**, **8**, **9**, **10**, **11**, **12**, **13**, **14**, **15**, **16**, **17**, **18**, **19**

*First generation polyester dendrimer***4**. Tris(4-hydroxybutyl) phosphate **3** (FW 314.3, 190 mg, 0.6 mmol), was dispersed in dry dichloromethane (6 mL). Dibenzyl trimesoate (**1a**) (800 mg, 2.05 mmol, 3.4 equiv.) and 1-ethyl-3-(3-dimethylaminopropyl)carbodiimide (EDC) (FW 191.7, 403 mg, 2.1 mmol, 3.5 equiv.) were added under argon atmosphere. The suspension was stirred vigorously and quickly dissolved. After 5 min, 4-dimethylaminopyridine (DMAP) (FW 122.2, 36 mg, 0.3 mmol, 0.4 equiv.) was added and the mixture was stirred at room temperature overnight. The reaction mixture was then diluted with ethyl acetate (60 mL), washed with 0.1 M citric acid (15 mL), dried (MgSO_4_), and evaporated under reduced pressure. The residue was placed on a short plug of silica gel. Elution with the CH_2_Cl_2_–MeOH 100:1 mixture and gradually increasing the polarity to CH_2_Cl_2_–MeOH 20:1, gave the title ester **4** (790 mg). Yield 92%. *R_f_* 0.3 (CH_2_Cl_2_-MeOH 20:1); NMR: δ_H_ (500 MHz, CDCl_3_) 1.86-1.87 [m, ^3^*J*(H,H) = 6.4 Hz, 6H, (POCH_2_CH_2_CH_2_CH_2_OC)], 1.91–1.92 [m, ^3^*J*(H,H) = 6.4 Hz, 6H, (POCH_2_CH_2_CH_2_CH_2_OC)], 4.14 [dt, ^3^*J*(H,H) = 5.8 Hz, ^3^*J*(P,H) = 11.2 Hz, 6H, (POCH_2_CH_2_CH_2_CH_2_OC)], 4.42 [t, ^3^*J*(H,H) = 5.8 Hz, 6H, (POCH_2_CH_2_CH_2_CH_2_OC)], 5.39 [s, 12H, (PhCH_2_)], 7.32–7.47 (m, 30H, Ph), 8.85 [d, ^4^*J*(H,H) = 1.5 Hz, 6H, Ar], 8.88 [t, ^4^*J*(H,H) = 1.5 Hz, 3H, Ar] ppm; δ_C_ (125 MHz, CDCl_3_) 25.05 [3C, O=COCH_2_CH_2_CH_2_CH_2_O)], 26.94 [d, ^3^*J*(C,P) = 6.3 Hz, 3C, (OCH_2_CH_2_CH_2_CH_2_OP)], 65.05 [3C, (OCH_2_CH_2_CH_2_CH_2_OP)], 67.20 [d, ^2^*J*(C,P) = 5.0 Hz, 3C, (OCH_2_CH_2_CH_2_CH_2_OP)], 67.43 [6C, (PhCH_2_)], 128.4 [12C, (PhCα)], 128.5 [6C, (PhCγ)], 128.7 [12C, (PhCβ)], 131.3 [6C, (*ipso* Ph)], 134.4 [3C, (Ar)], 134.7 [6C, (Ar)], 135.5 [9C, (*ipso* Ar)], 164.8 [6C, (C=O)], 164.9 [3C, (C=O)] ppm; δ_P_ {^1^H} (202 MHz, CDCl_3_) −0.51 ppm; MALDI TOF MS calcd. for C_81_H_75_O_22_P, M = 1430.4. Found *m*/*z* = 1453.9 M + Na, 1469.9 M + K.

*Polyanionic dendrimer***6**. Dendrimer **4** (FW 1431.4, 300 mg, 0.21 mmol) was dissolved in methanol (4 mL) and palladium on activated charcoal (10%, 100 mg) was then added. The reaction mixture was stirred under a hydrogen atmosphere for 10 h. The catalyst was filtered off, the filtrate concentrated to dryness in vacuo to furnish analytically pure acid **5** (181 mg, 97%) as a white solid. NMR: δ_H_ (500 MHz, CDCl_3_:CD_3_OD 1:1) 1.73-1.74 [m, ^3^*J*(H,H) = 6.4 Hz, 6H, (POCH_2_CH_2_CH_2_CH_2_OC)], 1.83–1.84 [m, ^3^*J*(H,H) = 6.4 Hz, 6H, (POCH_2_CH_2_CH_2_CH_2_OC)], 4.09 [dt, ^3^*J*(H,H) = 5.8 Hz, ^3^*J*(P,H) = 11.2 Hz, 6H, (POCH_2_CH_2_CH_2_CH_2_OC)], 4.31 [t, ^3^*J*(H,H) = 5.8 Hz, 6H, (POCH_2_CH_2_CH_2_CH_2_OC)], 8.71 [d, ^4^*J*(H,H) = 1.6 Hz, 6H, Ar], 8.74 [t, ^4^*J*(H,H) = 1.6 Hz, 3H, Ar] ppm; δ_C_ {^1^H, ^31^P} (125 MHz, CD_3_OD-CDCl_3_ 2:1) 24.35 [3C, O=COCH_2_CH_2_CH_2_CH_2_O)], 26.00 [3C, (OCH_2_CH_2_CH_2_CH_2_OP)], 64.45 [3C, (OCH_2_CH_2_CH_2_CH_2_OP)], 67.07 [3C, (OCH_2_CH_2_CH_2_CH_2_OP)], 130.4 [3C, (*ipso* Ar)], 131.2 [6C, (*ipso* Ar)], 133.5 [6C, (Ar)], 134.0 [3C, (Ar)], 164.5 [3C, (C=O)], 166.0 [6C, (C=O)] ppm; δ_P_ {^1^H} (202 MHz, CD_3_OD-CDCl_3_ 2:1) −0.42 ppm; MALDI TOF MS calcd. for C_39_H_39_O_22_P, M = 890.2. Found *m*/*z* = 890.2. Next, acid **5** (60 mg, 0.066 mmol) was dispersed in water (2 mL) and solid sodium bicarbonate (34 mg, 0.4 mmol, 6 equiv.) was added. After 20 min, the resulting solution was frozen and lyophilized under high vacuum (0.1 mmHg) to give **6** as a hexasodium salt of **5** (68 mg), as a nonhygroscopic white powder.

*Second generation polyester dendrimer***7**. Carboxy-terminated, first generation phosphate dendrimer **5** (FW 890.7, 100 mg, 0.112 mmol), was dissolved in dry THF (2 mL). Then, the reaction mixture was diluted with dry dichloromethane (2 mL). Di-*tert*-butyl 5-hydroxymethylbenzene-1,3-dicarboxylate (**2**) (FW 308.4, 230 mg, 0.74 mmol, 6.6 equiv.) and 1-ethyl-3-(3-dimethylaminopropyl)carbodiimide (EDC) (172 mg, 0.896 mmol, 8.0 equiv.) were added under argon atmosphere. The suspension was stirred vigorously and quickly dissolved. After 5 min, 4-dimethylaminopyridine (DMAP) (15 mg, 0.12 mmol, 1.0 equiv.) was added and the mixture was stirred at room temperature overnight. The reaction mixture was then diluted with ethyl acetate (50 mL), washed with 0.1 M citric acid (10 mL), dried (MgSO_4_), and evaporated under reduced pressure. The residue was placed on a short plug of silica gel. Elution with the cyclohexane–acetone 20:1 mixture and gradually increasing the polarity to cyclohexane–acetone 8:1, gave the title ester **5** (260 mg). Yield 88%. NMR: δ_H_ (500 MHz, CDCl_3_-C_6_D_6_ 2:1) 1.58 [s, 108H, C(CH_3_)_3_], 1.81–1.86 [m, 6H, (POCH_2_CH_2_CH_2_CH_2_OC)], 1.87–1.96 [m, 6H, (POCH_2_CH_2_CH_2_CH_2_OC)], 4.13 [dt, ^3^*J*(H,H) = 6.3 Hz, ^3^*J*(P,H) = 12.3 Hz, 6H, (POCH_2_CH_2_CH_2_CH_2_OC)], 4.40 [t, ^3^*J*(H,H) = 6.5 Hz, 6H, (POCH_2_CH_2_CH_2_CH_2_OC)], 5.29 [s, 12H, (ArCH_2_)], 8.20 [d, ^4^*J*(H,H) = 1.3 Hz, 12H, Ar^2^], 8.53 [t, ^4^*J*(H,H) = 1.3 Hz, 6H, Ar^2^], 8.85 [d, ^3^*J*(H,H) = 1.5 Hz, 6H, Ar^1^], 8.87 [t, ^3^*J*(H,H) = 1.5 Hz, 3H, Ar^1^] ppm; δ_C_ (125 MHz, CDCl_3_) 25.04 [3C, O=COCH_2_CH_2_CH_2_CH_2_O)], 26.96 [d, ^3^*J*(C,P) = 7.1 Hz, 3C, (OCH_2_CH_2_CH_2_CH_2_OP)], 28.21 [36C, C(CH_3_)_3_], 65.16 [3C, (OCH_2_CH_2_CH_2_CH_2_OP)], 67.43 [6C, (ArCH_2_)], 67.20 [d, ^2^*J*(C,P) = 5.7 Hz, 3C, (OCH_2_CH_2_CH_2_CH_2_OP)], 81.92 [12C, C(CH_3_)_3_], 130.6 [6C, (Ar^2^)], 131.1 [6C, (*ipso* Ar^1^)], 131.5 [3C, (*ipso* Ar^1^)], 132.9 [12C, (*ipso* Ar^2^)], 133.2 [12C, (Ar^2^)], 134.9 [3C, (Ar^1^)], 135.0 [6C, (Ar^1^)], 136.1 [6C, (*ipso* Ar^2^)], 164.7 [18C, (C=O)], 164.8 [3C, (C=O)] ppm; δ_P_ {^1^H} (202 MHz, CDCl_3_) 0.53 ppm; MALDI TOF MS calcd. for C_141_H_171_O_46_P, M = 2631.1. Found *m*/*z* = 2670.3 M + K.

*Polyanionic dendrimer***9**. Dendrimer **7** (FW 2632.8, 134 mg, 0.05 mmol) was dissolved in dry dichloromethane (2 mL), and trifluoroacetic acid was added (2 mL). The reaction mixture was stirred at room temperature under argon atmosphere for 5 h. All the volatiles were removed *in vacuo*, and the residue was dissolved in THF (1 mL). Next, acetone (10 mL) was added to that solution and the resulting mixture was kept in the refrigerator for about an hour. The precipitate was filtered off to provide acid **8** (88 mg, 90%) as a white solid. NMR: δ_H_ (500 MHz, CDCl_3_-CD_3_OD 2:1) 2.03–2.08 [m, 6H, (POCH_2_CH_2_CH_2_CH_2_OC)], 2.25–2.28 [m, 6H, (POCH_2_CH_2_CH_2_CH_2_OC)], 3.90-3.92 [m, 6H, (POCH_2_CH_2_CH_2_CH_2_OC)], 4.14–4.16 [m, 6H, (POCH_2_CH_2_CH_2_CH_2_OC)], 5.22 [s, 12H, (ArCH_2_)], 8.36 [s, 6H, Ar^2^], 8.48 [s, 12H, Ar^2^], 8.56 [s, 6H, Ar^1^], 8.61 [s, 3H, Ar^1^] ppm; δ_P_ {^1^H} (202 MHz, CD_3_OD-CDCl_3_ 2:1) −2.80 ppm; MALDI TOF MS calcd. for C_93_H_75_O_46_P, M = 1959.3. Found *m*/*z* = 1982.6 M + Na. Later on, acid **8** (35 mg, 0.0179 mmol) was dispersed in water (2 mL) and solid sodium bicarbonate (18 mg, 0.2 mmol, 12 equiv.) was added. After 20 min, the resulting solution was frozen and lyophilized under high vacuum (0.1 mmHg) to give **9** as a dodecasodium salt of **8** (40 mg), as a nonhygroscopic white powder.

*Third generation polyester dendrimer***10**. Carboxy-terminated, second generation phosphate dendrimer **8** (FW 1959.5, 40 mg, 0.02 mmol), was dissolved in dry DMF (1.5 mL). Then, the resulting solution was diluted with dry dichloromethane (2 mL). Di-*tert*-butyl 5-hydroxymethylbenzene-1,3-dicarboxylate (**2**) (FW 308.4, 82 mg, 0.264 mmol, 13.2 equiv.) and 1-ethyl-3-(3-dimethylaminopropyl)carbodiimide (EDC) (172 mg, 0.3 mmol, 15 equiv.) were added under argon atmosphere. The suspension was stirred vigorously and quickly dissolved. After 5 min, 4-dimethylaminopyridine (DMAP) (5 mg, 0.04 mmol, 1.8 equiv.) was added and the mixture was stirred at room temperature overnight. The reaction mixture was then diluted with ethyl acetate (50 mL), washed with 0.1 M citric acid (10 mL), dried (MgSO_4_), and evaporated under reduced pressure. The residue was placed on a short plug of silica gel. Elution with the cyclohexane–acetone 20:1 mixture and gradually increasing the polarity to cyclohexane–acetone 6:1, provided the title ester **10** (83 mg). Yield 76%. NMR: δ_H_ (500 MHz, CDCl_3_) 1.56 [s, 216H, C(CH_3_)_3_] 1.82–1.87 [m, 6H, (POCH_2_CH_2_CH_2_CH_2_OC)], 1.88–1.95 [m, 6H, (POCH_2_CH_2_CH_2_CH_2_OC)], 4.13 [dt, ^3^*J*(H,H) = 5.9 Hz, ^3^*J*(P,H) = 12.2 Hz, 6H, (POCH_2_CH_2_CH_2_CH_2_OC)], 4.39 [t, ^3^*J*(H,H) = 6.4 Hz, 6H, (POCH_2_CH_2_CH_2_CH_2_OC)], 5.44 [s, 24H, (Ar^3^CH_2_)], 5.46 [s, 12H, (Ar^2^CH_2_)], 8.19 [s, 24H, Ar^3^], 8.35 [s, 12H, Ar^3^], 8.51 [s, 12H, Ar^2^], 8.69 [s, 6H, Ar^2^], 8.83 [s, 6H, Ar^1^], 8.85 [s, 3H, Ar^1^] ppm; δ_C_ (125 MHz, CDCl_3_) 24.94 [3C, (O=COCH_2_CH_2_CH_2_CH_2_O)], 26.91 [d, ^3^*J*(C,P) = 7.4 Hz, 3C, (OCH_2_CH_2_CH_2_CH_2_OP)], 28.14 [72C, C(CH_3_)_3_], 65.12 [3C, (OCH_2_CH_2_CH_2_CH_2_OP)], 66.27 [6C, (Ar^2^CH_2_)], 66.33 [12C, (Ar^3^CH_2_)], 67.09 [d, ^2^*J*(C,P) = 6.7 Hz, 3C, (OCH_2_CH_2_CH_2_CH_2_OP)], 81.81 [24C, C(CH_3_)_3_], 130.5 [12C, (Ar^3^)], 131.0 [6C, (Ar^2^)], 133.0 [24C, (Ar^3^)], 130.5 [12C, (Ar^3^)], 130.8 [6C, (*ipso* Ar^1^)], 131.0 [12C, (*ipso* Ar^2^)], 131.5 [3C, (*ipso* Ar^1^)], 132.8 [24C, (*ipso* Ar^3^)], 134.2 [12C, (Ar^2^)], 135.0 [9C, (Ar^1^)], 136.2 [12C, (*ipso* Ar^3^)], 136.7 [6C, (*ipso* Ar^2^)], 164.5 [9C, (C^1^=O)], 164.6 [24C, (C^3^=O)], 165.0 [12C, (C^2^=O)] ppm; δ_P_ {^1^H} (202 MHz, CDCl_3_) -0.46 ppm. MALDI TOF MS calcd. for C_297_H_339_O_94_P, M = 5440.1. Found *m*/*z* = 5477.8 M + K.

*Polyanionic dendrimer***12**. Dendrimer **10** (80 mg, 0.015 mmol) was dissolved in dry dichloromethane (1.5 mL), and trifluoroacetic acid was added (2 mL). The reaction mixture was stirred at room temperature under argon atmosphere for 5 h. All the volatiles were removed *in vacuo*, and the residue was dissolved in THF (1 mL). Next, acetone (10 mL) was added to that solution and the resulting mixture was kept in the refrigerator for about an hour. The precipitate was filtered off to provide acid **11** (54 mg, 88%) as a white solid. NMR: δ_H_ (500 MHz, THF-d_8_) 1.75–1.80 [m, 6H, (POCH_2_CH_2_CH_2_CH_2_OC)], 1.82–1.88 [m, ^3^*J*(H,H) = 6.4 Hz, 6H, (POCH_2_CH_2_CH_2_CH_2_OC)], 4.07 [dt, ^3^*J*(P,H) = 12.3 Hz, ^3^*J*(H,H) = 6.2 Hz, 6H, (POCH_2_CH_2_CH_2_CH_2_OC)], 4.33 [t, ^3^*J*(H,H) = 6.2 Hz, 6H, (POCH_2_CH_2_CH_2_CH_2_OC)], 5.48 [s, 24H, (Ar^3^CH_2_)], 5.49 [s, 12H, (Ar^2^CH_2_)], 8.30 [d, ^4^*J*(H,H) = 1.3 Hz, 33H, 9HAr^1^, 24HAr^3^], 8.58 [s, 12H, Ar^3^], 8.76 [d, ^4^*J*(H,H) = 1.6 Hz, 12H, Ar^2^], 8.81 [t, ^4^*J*(H,H) = 1.5 Hz, 6H, Ar^2^] ppm; δ_C_ (125 MHz, THF-d_8_) 26.60 [d, ^3^*J*(C,P) = 7.2 Hz, 3C, (OCH_2_CH_2_CH_2_CH_2_OP)], 29.24 [3C, (O=COCH_2_CH_2_CH_2_CH_2_O)], 64.84 [6C, (Ar^2^CH_2_)], 66.08 [12C, (Ar^3^CH_2_)], 67.09 [3C, (OCH_2_CH_2_CH_2_CH_2_OP)], 67.34 [d, ^2^*J*(C,P) = 6.7 Hz, 3C, (OCH_2_CH_2_CH_2_CH_2_OP)], 130.6 [12C, (Ar^3^)], 131.1 [12C, (Ar^2^)], 131.4 [6C, (Ar^2^)], 131.7 [24C, (*ipso* Ar^3^)], 133.3 [12C, (*ipso* Ar^2^)], 133.3 [6C, (Ar^1^)], 133.4 [24C, (Ar^3^)], 133.6 [3C, (Ar^1^)], 134.1 [6C, (*ipso* Ar^2^)], 134.2 [12C, (*ipso* Ar^3^)], 136.8 [9C, (Ar^1^)], 164.0 [12C, (C^2^=O)], 164.1 [9C, (C^1^=O)], 165.9 [24C, (C^3^=O)] ppm; δ_P_ {^1^H} (202 MHz, THF-d_8_) −0.95 ppm; MALDI TOF MS calcd. for C_201_H_147_O_94_P, M = 4096.7.3. Found *m*/*z*, fragmentation: 4129.6, 3965.6, 3949.9, 3844.2, 3731.4, 3700.0, 3601.9 (major peaks). Elemental analysis: Found (acid): C, 58.87; H, 3.66; C_201_H_147_O_94_P requires C, 58.92; H, 3.62; P, 0.76%. Afterwards, acid **11** (FW 4097.2, 40 mg, 0.0098 mmol) was dispersed in water (2 mL) and solid sodium bicarbonate (20 mg, 0.235 mmol, 24 equiv.) was added. After 20 min, the resulting solution was frozen and lyophilized under high vacuum (0.1 mmHg) to give **12** as a tetracosasodium salt of **11** (45 mg), as a nonhygroscopic white powder.

*First generation polyester thiophosphate dendrimer***14**. Tris(4-hydroxybutyl) thiophosphate **13** (FW 330.4, 166 mg, 0.5 mmol), was dissolved in dry dichloromethane (5 mL). Di-*tert*-butyl trimesoate (**1**) (322.4, 560 mg, 1.7 mmol, 3.4 equiv.) and 1-ethyl-3-(3-dimethylaminopropyl)carbodiimide (EDC) (FW 191.7, 336 mg, 1.75 mmol, 3.5 equiv.) were added under argon atmosphere. The suspension was stirred vigorously and quickly dissolved. After 5 min, 4-dimethylaminopyridine (DMAP) (FW 122.2, 25 mg, 0.2 mmol, 0.4 equiv.) was added and the mixture was stirred at room temperature overnight. The reaction mixture was then diluted with ethyl acetate (60 mL), washed with 0.1 M citric acid (15 mL), dried (MgSO_4_), and evaporated under reduced pressure. The residue was placed on a short plug of silica gel. Elution with the cyclohexane–acetone 100:1 mixture and gradually increasing the polarity to cyclohexane–acetone 20:1, gave the title ester **14** (560 mg). Yield 90%. *R_f_* 0.25 (cyclohexane–acetone 25:1). NMR: δ_H_ (500 MHz, CDCl_3_) 1.59 [s, 54H, C(CH_3_)_3_], 1.79-1.84 [m, ^3^*J*(H,H) = 6.4 Hz, 12H, (POCH_2_CH_2_CH_2_CH_2_OC) and (POCH_2_CH_2_CH_2_CH_2_OC)], 4.12 [dt, ^3^*J*(H,H) = 5.8 Hz, ^3^*J*(P,H) = 12.6 Hz, 6H, (POCH_2_CH_2_CH_2_CH_2_OC)], 4.42 [t, ^3^*J*(H,H) = 5.7 Hz, 6H, (POCH_2_CH_2_CH_2_CH_2_OC)], 8.70 [s, 3H, Ar], 8.71 [s, 6H, Ar] ppm; δ_C_ (125 MHz, CDCl_3_) 25.13 [3C, O=COCH_2_CH_2_CH_2_CH_2_O)], 26.77 [d, ^3^*J*(C,P) = 7.8 Hz, 3C, (OCH_2_CH_2_CH_2_CH_2_OP)], 28.19 [18C, C(CH_3_)_3_], 64.93 [3C, (OCH_2_CH_2_CH_2_CH_2_OP)], 67.70 [d, ^2^*J*(C,P) = 5.4 Hz, 3C, (OCH_2_CH_2_CH_2_CH_2_OP)], 82.24 [6C, C(CH_3_)_3_], 130.9 [3C, (*ipso* Ar)], 132.8 [6C, (*ipso* Ar)], 134.0 [6C, (Ar)], 134.4 [3C, (Ar)], 164.3 [6C, (C=O)], 165.3 [3C, (C=O)] ppm; δ_P_ {^1^H} (202 MHz, CDCl_3_) 68.52 ppm; MALDI TOF MS calcd. for C_63_H_87_O_21_PS, M = 1242.5. Found *m*/*z* = 1282.5 M + K.

*Polyanionic dendrimer***16**. Dendrimer **14** (FW 1243.4, 125 mg, 0.1 mmol) was dissolved in dry dichloromethane (1.5 mL), and trifluoroacetic acid was added (2 mL). The reaction mixture was stirred at room temperature under argon atmosphere for 5 h. All the volatiles were removed *in* *vacuo*, and the residue was dissolved in THF (1 mL). Next, acetone (10 mL) was added to that solution and the resulting mixture was kept in the refrigerator for about an hour. The precipitate was filtered off to provide acid **15** (84 mg, 93%) as a white solid. NMR: δ_H_ (500 MHz, CDCl_3_-THF-d_8_ 1:5) 1.60–1.69 [m, 12H, (POCH_2_CH_2_CH_2_CH_2_OC) and (POCH_2_CH_2_CH_2_CH_2_OC)], 3.86 [dt, ^3^*J*(H,H) = 6.0 Hz, ^3^*J*(P,H) = 12.6 Hz, 6H, (POCH_2_CH_2_CH_2_CH_2_OC)], 4.11 [t, ^3^*J*(H,H) = 6.0 Hz, 6H, (POCH_2_CH_2_CH_2_CH_2_OC)], 8.52 [s, 6H, Ar], 8.55 [s, 3H, Ar] ppm; δ_C_ (125 MHz, CDCl_3_-THF-d_8_ 1:5) 25.06 [3C, O=COCH_2_CH_2_CH_2_CH_2_O)], 26.22 [d, ^3^*J*(C,P) = 7.5 Hz, 3C, (OCH_2_CH_2_CH_2_CH_2_OP)], 67.04 [d, ^2^*J*(C,P) = 5.2 Hz, 3C, (OCH_2_CH_2_CH_2_CH_2_OP)], 67.25 [3C, (OCH_2_CH_2_CH_2_CH_2_OP)], 130.6 [9C, (*ipso* Ar)], 131.3 [9C, (Ar)], 164.3 [3C, (C=O)], 165.6 [6C, (C=O)] ppm; δ_P_ {^1^H} (202 MHz, CDCl_3_-THF-d_8_ 1:5) 68.52 ppm; HRMS (TOF MS ES) M − H^+^: found 905.1371. C_39_H_39_O_21_PS requires 905.1364. Soon after, acid **15** (35 mg, 0.0179 mmol) was dispersed in water (2 mL) and solid sodium bicarbonate (10 mg, 0.11 mmol, 6 equiv.) was added. After 20 min, the resulting solution was frozen and lyophilized under high vacuum (0.1 mmHg) to give **16** as a hexasodium salt of **15** (40 mg), as a nonhygroscopic white powder.

*Second generation polyester thiophosphate dendrimer***17**. Carboxy-terminated, first generation thiophosphate dendrimer **15** (FW 906.8, 40 mg, 0.044 mmol) was dissolved in dry THF (1.0 mL). Then, the reaction mixture was diluted with dry dichloromethane (2 mL). Di-*tert*-butyl 5-hydroxymethylbenzene-1,3-dicarboxylate (**2**) (FW 308.4, 67 mg, 0.3 mmol, 6.6 equiv.) and 1-ethyl-3-(3-dimethylaminopropyl)carbodiimide (EDC) (70 mg, 0.36 mmol, 8.0 equiv.) were added under argon atmosphere. The suspension was stirred vigorously and quickly dissolved. After 5 min, 4-dimethylaminopyridine (DMAP) (FW 122.2, 5.0 mg, 0.04 mmol, 0.9 equiv.) was added and the mixture was stirred at room temperature overnight. The reaction mixture was then diluted with ethyl acetate (20 mL), washed with 0.1 M citric acid (10 mL), dried (MgSO_4_), and evaporated under reduced pressure. The residue was placed on a short plug of silica gel. Elution with the cyclohexane–acetone 100:1 mixture and gradually increasing the polarity to cyclohexane–acetone 15:1, afforded the title ester **17** (95 mg). Yield 84%. δ_H_ (500 MHz, CDCl_3_) 1.58 [s, 108H, C(CH_3_)_3_], 1.80–1.87 [m, 6H, (POCH_2_CH_2_CH_2_CH_2_OC)], 1.88–1.96 [m, 6H, (POCH_2_CH_2_CH_2_CH_2_OC)], 4.12 [dt, ^3^*J*(H,H) = 6.3 Hz, ^3^*J*(P,H) =14.3 Hz, 6H, (POCH_2_CH_2_CH_2_CH_2_OC)], 4.40 [t, ^3^*J*(H,H) = 6.4 Hz, 6H, (POCH_2_CH_2_CH_2_CH_2_OC)], 5.46 [s, 12H, (ArCH_2_)], 8.20 (s, 12H, Ar^2^), 8.53 (s, 6H, Ar^2^), 8.85 (s, 3H, Ar^1^), 8.87 (s, 6H, Ar^1^) ppm; δ_C_ (125 MHz, CDCl_3_) 25.01 [3C, O=COCH_2_CH_2_CH_2_CH_2_O)], 26.66 [d, ^3^*J*(C,P) = 7.6 Hz, 3C, (OCH_2_CH_2_CH_2_CH_2_OP)], 28.16 [36C, C(CH_3_)_3_], 65.13 [3C, (OCH_2_CH_2_CH_2_CH_2_OP)], 66.51 [6C, (ArCH_2_)], 67.61 [d, ^2^*J*(C,P) = 5.7 Hz, 3C, (OCH_2_CH_2_CH_2_CH_2_OP)], 81.87 [12C, C(CH_3_)_3_], 130.6 [6C, (Ar^2^)], 130.9 [6C, (*ipso* Ar^1^)], 131.6 [3C, (*ipso* Ar^1^)], 132.8 [12C, (*ipso* Ar^2^)], 133.1 [12C, (Ar^2^)], 134.9 [3C, (Ar^1^)], 135.0 [6C, (Ar^1^)], 136.0 [6C, (*ipso* Ar^2^)], 164.6 [21C, (C=O)] ppm; δ_P_ {^1^H} (202 MHz, CD_3_OD-CDCl_3_ 2:1) 68.60 ppm; MALDI TOF MS calcd. for C_141_H_171_O_45_PS, M = 2647.0. Found *m*/*z* = 2670.4 M + Na, 2686.7 M + K.

*Polyanionic dendrimer***19**. Dendrimer **17** (FW 2648.9, 70 mg, 0.0264 mmol) was dissolved in dry dichloromethane (1.5 mL), and trifluoroacetic acid was added (1.5 mL). The reaction mixture was stirred at room temperature under argon atmosphere for 5 h. All the volatiles were removed *in vacuo*, and the residue was dissolved in THF (1 mL). Next, acetone (10 mL) was added to that solution and the resulting mixture was kept in the refrigerator for about an hour. The precipitate was filtered off to provide acid **18** (48 mg, 92%) as a white solid. NMR: δ_H_ (500 MHz, THF-d_8_) 1.82–1.88 [m, 6H, (POCH_2_CH_2_CH_2_CH_2_OC)], 1.90-1.98 [m, 6H, (POCH_2_CH_2_CH_2_CH_2_OC)], 3.59-3.64 [m, 6H, (POCH_2_CH_2_CH_2_CH_2_OC)], 3.80 [t, ^3^*J*(H,H) = 6.8 Hz, 6H, (POCH_2_CH_2_CH_2_CH_2_OC)], 5.47 [s, 12H, (ArCH_2_)], 8.30 (s, 12H, Ar^2^), 8.36 (s, 6H, Ar^2^), 8.54 (s, 6H, Ar^1^), 8.61 (s, 3H, Ar^1^), 10.87 (brs, 12H, COOH) ppm; δ_C_ (125 MHz, THF-d_8_) 25.40 [3C, O=COCH_2_CH_2_CH_2_CH_2_O)], 28.96 [3C, (OCH_2_CH_2_CH_2_CH_2_OP)], 65.92 [3C, (OCH_2_CH_2_CH_2_CH_2_OP)], 66.96, [3C, (OCH_2_CH_2_CH_2_CH_2_OP)], 67,24 [6C, (ArCH_2_)], 128.6 [9C, (Ar^1^)], 130.5 [12C, (*ipso* Ar^2^)], 130.9 [6C, (*ipso* Ar^2^)], 131.6 [6C, (Ar^2^)], 133.5 [12C, (Ar^2^)], 137.1 [9C, (*ipso* Ar^1^)], 163.9 [3C, (C=O)], 164.5 [6C, (C=O)], 165.8 [12C, (HOC=O)] ppm δ_P_ {^1^H} (202 MHz, THF-d_8_) 68.71 ppm; MALDI TOF MS calcd. for C_93_H_75_O_45_PS, M = 1974.3. Found *m*/*z*, fragmentation: 1149.8, 1121.7, 1093.5, 1065.5 (major peaks). Elemental analysis: Found (acid): C, 56.51; H, 3.85; S, 1.62%. C_93_H_75_O_45_PS requires C, 56.54; H, 3.83; S, 1.62%. Afterwards, acid **18** (35 mg, 0.0179 mmol) was dispersed in water (2 mL) and solid sodium bicarbonate (18 mg, 0.2 mmol, 12 equiv.) was added. After 20 min, the resulting solution was frozen and lyophilized under high vacuum (0.1 mmHg) to give **19** as a dodecasodium salt of **18** (40 mg), as a nonhygroscopic white powder.

*Second generation fully protected dendrimer***21**. Hydroxy-terminated, first generation thiophosphate dendrimer^12^ **20** (FW 1009.1, 51 mg, 0.050 mmol) was suspended in dry dichloromethane (3.0 mL). Di-*tert*-butyl 1.3.5-benzenetricarboxylate (**1**) (FW 322.4, 105 mg, 0.325 mmol, 6.5 equiv.) and 1-ethyl-3-(3-dimethylaminopropyl)carbodiimide (EDC) (77 mg, 0.4 mmol, 8.0 equiv.) were added under argon atmosphere. The suspension was stirred vigorously and quickly dissolved. After 5 min, 4-dimethylaminopyridine (DMAP) (FW 122.2, 5.0 mg, 0.04 mmol, 0.9 equiv.) was added and the mixture was stirred at room temperature overnight. The reaction mixture was then diluted with ethyl acetate (20 mL), washed with 0.1 M citric acid (10 mL), dried (MgSO_4_), and evaporated under reduced pressure. The residue was placed on a short plug of silica gel. Elution with the neat dichloromethane provided the title dendrimer **21** (127 mg). Yield 90%. δ_H_ (500 MHz, CDCl_3_) 1.58 [s, 108H, C(CH_3_)_3_], 1.78–1.87 [m, 12H, (POCH_2_CH_2_CH_2_CH_2_OC)], 1.88–1.95 [m, 12H, (POCH_2_CH_2_CH_2_CH_2_OC)], 2.12–2.21 [m, 6H, (POCH_2_CH_2_CH_2_OP)], 4.12 [dt, ^3^*J*(H,H) = 6.3 Hz, ^3^*J*(P,H) = 13.6 Hz, 12H, (POCH_2_CH_2_CH_2_CH_2_OC)], 4.24 [dt, ^3^*J*(H,H) = 6.3 Hz, ^3^*J*(P,H) = 14.3 Hz, 12H, (POCH_2_CH_2_CH_2_OP)], 4.45 [t, ^3^*J*(H,H) = 6.1 Hz, 12H, (POCH_2_CH_2_CH_2_CH_2_OC)], 8.70 (s, 6H, Ar), 8.71 (s, 12H, Ar) ppm; δ_C_ (125 MHz, CDCl_3_), 25.07 [6C, O=COCH_2_CH_2_CH_2_CH_2_O)], 26.71 [d, ^3^*J*(C,P) = 7.8 Hz, 6C, (OCH_2_CH_2_CH_2_CH_2_OP)], 28.13 [36C, C(CH_3_)_3_], 29.41–29.49 [m, ^3^*J*(C,P) = 7.8 Hz, 3C, (POCH_2_CH_2_CH_2_OP)], 61.62–61.73 [m, 6C, (POCH_2_CH_2_CH_2_OP)], 64.87 [6C, (OCH_2_CH_2_CH_2_CH_2_OP)], 67.64 [d, ^2^*J*(C,P) = 5.4 Hz, 6C, (OCH_2_CH_2_CH_2_CH_2_OP)], 82.17 [12C, C(CH_3_)_3_], 130.9 [6C, (*ipso* Ar)], 132.8 [12C, (*ipso* Ar)], 134.0 [12C, (Ar)], 134.4 [6C, (Ar)], 164.2 [12C, (C=O)], 165.2 [6C, (C=O)] ppm; δ_P_ {^1^H} (202 MHz, CDCl_3_), 68.29 (P^0^), 68.58 (3P^1^) ppm; MALDI TOF MS calcd. for C_135_H_192_O_48_P_4_S_4_, M = 2833.0. Found *m*/*z* = 2845.8 M + Na.

*Polyanionic dendrimer***23**. Dendrimer **21** (80 mg, 0.028 mmol) was dissolved in dry dichloromethane (1.5 mL), and trifluoroacetic acid was added (1.5 mL). The reaction mixture was stirred at room temperature under argon atmosphere for 5 h. All the volatiles were removed *in vacuo*, and the residue was dissolved in THF (1 mL). Next, acetone (11 mL) was added to that solution and the resulting mixture was kept in the refrigerator for about an hour. The precipitate was filtered off to provide acid **22** (56 mg, 93%) as a white solid. NMR: δ_H_ (200 MHz, CD_3_OD) 1.75–1.96 [m, 24H, (POCH_2_CH_2_CH_2_CH_2_OC)], 2.12–2.21 [m, 6H, (POCH_2_CH_2_CH_2_OP)], 4.09–4.21 [m, ^3^*J*(H,H) = 6.3 Hz, 12H, (POCH_2_CH_2_CH_2_CH_2_OC)], 4.24–4.30 [m, 12H, (POCH_2_CH_2_CH_2_OP)], 4.37 [t, ^3^*J*(H,H) = 6.0 Hz, 12H, (POCH_2_CH_2_CH_2_CH_2_OC)], 8.70 (s, 12H, Ar), 8.74 (s, 6H, Ar) ppm; δ_C_ (125 MHz, CD_3_OD-CDCl_3_ 3:1), 26.44 [6C, O=COCH_2_CH_2_CH_2_CH_2_O)], 28.09 [d, ^3^*J*(C,P) = 7.4 Hz, 6C, (OCH_2_CH_2_CH_2_CH_2_OP)], 30.60–30.70 [m, 3C, (POCH_2_CH_2_CH_2_OP)], 66.27–66.40 [m, 6C, (POCH_2_CH_2_CH_2_OP)], 66.53 [6C, (OCH_2_CH_2_CH_2_CH_2_OP)], 69.15 [d, ^2^*J*(C,P) = 5.6 Hz, 6C, (OCH_2_CH_2_CH_2_CH_2_OP)], 132.5 [6C, (*ipso* Ar)], 133.3 [6C, (Ar)], 135.6 [12C, (Ar)], 136.1 [12C, (*ipso* Ar)], 166.6 [6C, (C=O)], 167.9 [12C, (C=O)] ppm; δ_P_ {^1^H} (202 MHz, CD_3_OD-CDCl_3_ 3:1), 68.61 (P^0^), 68.80 (3P^1^) ppm; MALDI TOF MS calcd. for C_87_H_96_O_48_P_4_S_4_, M = 2160.3. Found *m*/*z*, fragmentation: 1470.0, 1450.8, 1005.7, 947.5, 930.7 (major peaks). Elemental analysis: Found (acid): C, 48.39; H, 4.41; S, 6.00%. C_87_H_96_O_48_P_4_S_4_ requires C, 48.34; H, 4.48; S, 5.93%. Finally, acid **22** (40 mg, 0.0185 mmol) was dispersed in water (2 mL) and solid sodium bicarbonate (19 mg, 0.22 mmol, 12 equiv.) was added. After 20 min, the resulting solution was frozen and lyophilized under high vacuum (0.1 mm Hg) to give **23** as a dodecasodium salt of **22** (47 mg), as a nonhygroscopic white powder.

## 4. Conclusions

In conclusion, using simple and readily available monomers, an easy and highly efficient method for the synthesis of new polyanionic dendrimers as potential broad-spectrum antiviral drugs in their own right was presented. The mild conditions of both the coupling and virtually quantitative deprotection reactions, provided highly pure and water-soluble macromolecular materials in good overall yields. For example, the total yields of the key compounds were as follows: Second generation acid **8**—64%, third generation polyacid **11**—42%, and second generation acid **18**—60%. The roughly estimated time of the preparation of each generation (two steps—condensation, purification, cleavage of the terminal esters, and purification) should fit easily within the 2-day-period. The important advantage of the presented strategy is direct access to the polyanionic material at each generation of the prepared dendrimer. Therefore, the synthesis is at least one difficult synthetic step shorter. This approach seems to be somehow a general methodology, which enables the transformation of practically any macromolecular compound terminated with hydroxy functions, into its polyanionic derivative. Moreover, it offers the possibility to make discrete modifications layer by layer (i.e., P=O, P=S, and/or carbon branching) within the same dendrimer skeleton, a key for a structure-activity relationship study.

Although the chemical synthesis of dendrimers is more than two decades old, the most significant reason hampering the broader use of dendrimers in biomedicine is usually difficult and their multistep preparation is time-consuming, especially for the high generation structures. Consequently, developing methodologies offering faster access to the important macromolecular material is especially warranted.

## Data Availability

The data presented in this study are available in the Supplementary Materials.

## Supplementary Materials

^1^H, ^31^P, ^13^C NMR, and mass spectra of the products synthesized in this work are available online.

## Sample Availability

Samples of the polyanionic dendrimers **6**, **9**, **12**, **16**, **19**, **23** are available from the author.
