# Supplementary material for: A Fast and Convenient Synthesis of New Water-Soluble, Polyanionic Dendrimers
Source: Molecules. 2021 Aug 6;26(16):4754. doi: 10.3390/molecules26164754 (PMC8399870; doi:10.3390/molecules26164754)

Supplementary Material

A fast and convenient synthesis of new water-soluble, polyanionic dendrimers.

Grzegorz M. Salamończyk

Centre of Molecular and Macromolecular Studies. The Polish Academy of Sciences, Sienkiewicza 112, 90-363 Łódź, Poland.
Tel.: + 48-42-684-7113; fax: +48-42-684-7126; e-mail: gmsalamo@cbmm.lodz.pl

The NMR and Mass spectra.

Spectra of the acid **1**..pages S2 – S3

Spectra of the benzyl-type alcohol **2**................................pages S3 – S4

Spectra of the dendrimer **4**..pages S5 - S7

Spectra of the dendrimer **5** (**6**) pages S8 - S9

Spectra of the dendrimer **7**..pages S10 - S12

Spectra of the dendrimer **8** (**9**) pages S13 - S14

Spectra of the dendrimer **10**..pages S15 - S18

Spectra of the dendrimer **11** (**12**) pages S18 - S21

Spectra of the dendrimer **14**..pages S21 – S23

Spectra of the dendrimer **15** (**16**) pages S24 - S25

Spectra of the dendrimer **17**..pages S26 – S28

Spectra of the dendrimer **18** (**19**) pages S28 – S30

Spectra of the dendrimer **21**..pages S31 – S32

Spectra of the dendrimer **22** (**23**) pages S33 – S35

Spectra of the acid **1**.

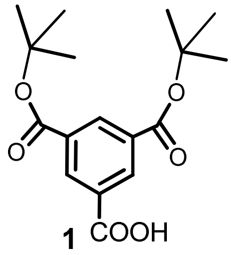

1

^{1}H NMR

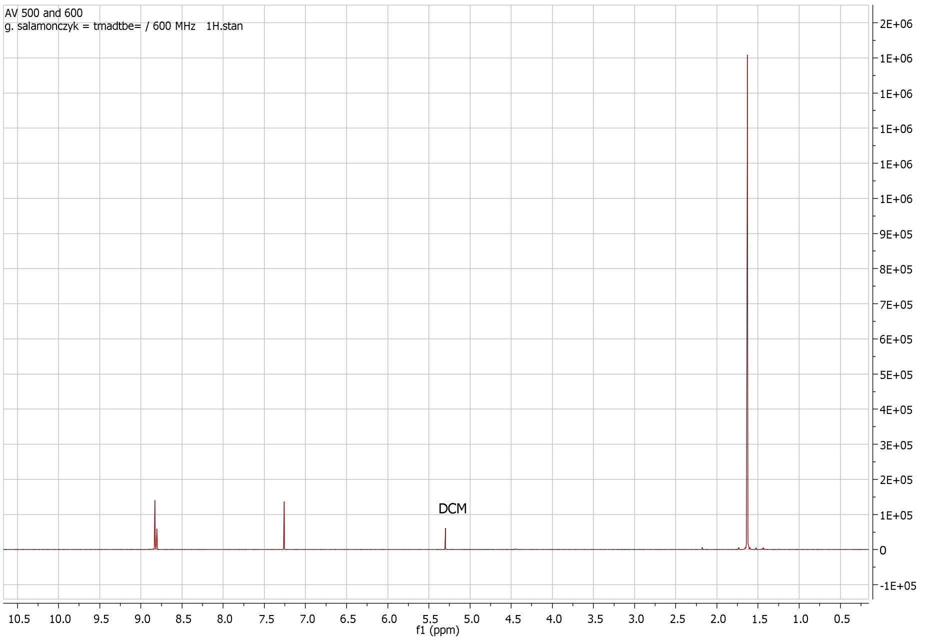

^{13}C NMR

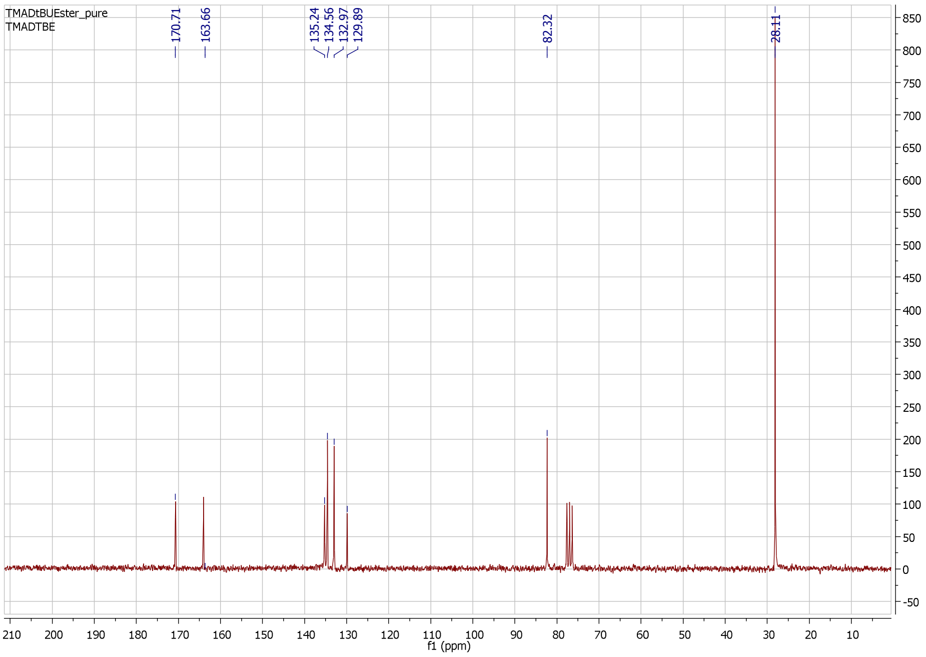

Mass Spectrum

Elemental Composition Report

Page 1

Single Mass Analysis
Tolerance = 5.0 PPM / DBE: min = -1.5, max = 70.0
Element prediction: Off
Number of isotope peaks used for i-FIT = 9

Monoisotopic Mass, Odd and Even Electron Ions
18 formula(e) evaluated with 1 results within limits (all results (up to 1000) for each mass)
Elements Used:
C: 0-30 H: 0-25 O: 0-7

Salamo
200203_TMADTBE_APCInA 16 (0.177) Cm (14:24-(3:11+41:72))

TOF MS AP-
2.34e+005

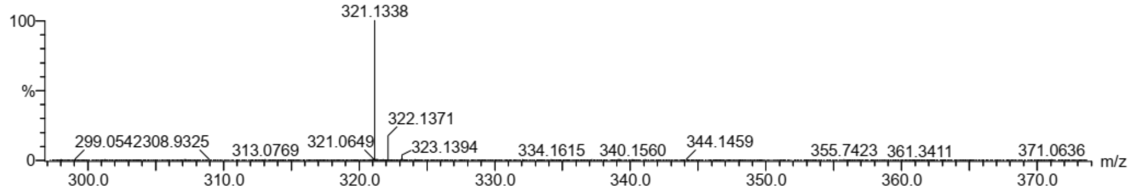

	Mass	Calc. Mass	mDa	PPM	DBE	i-FIT	Norm	Conf(%)	Formula
Minimum:					-1.5				
Maximum:			5.0	5.0	70.0				
	321.1338	321.1338	0.0	0.0	7.5	1539.8	n/a	n/a	C17 H21 O6

Spectra of the benzyl-type alcohol **2**

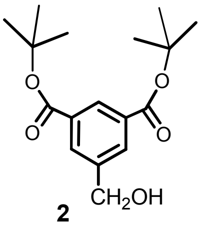

2

^{1}H NMR

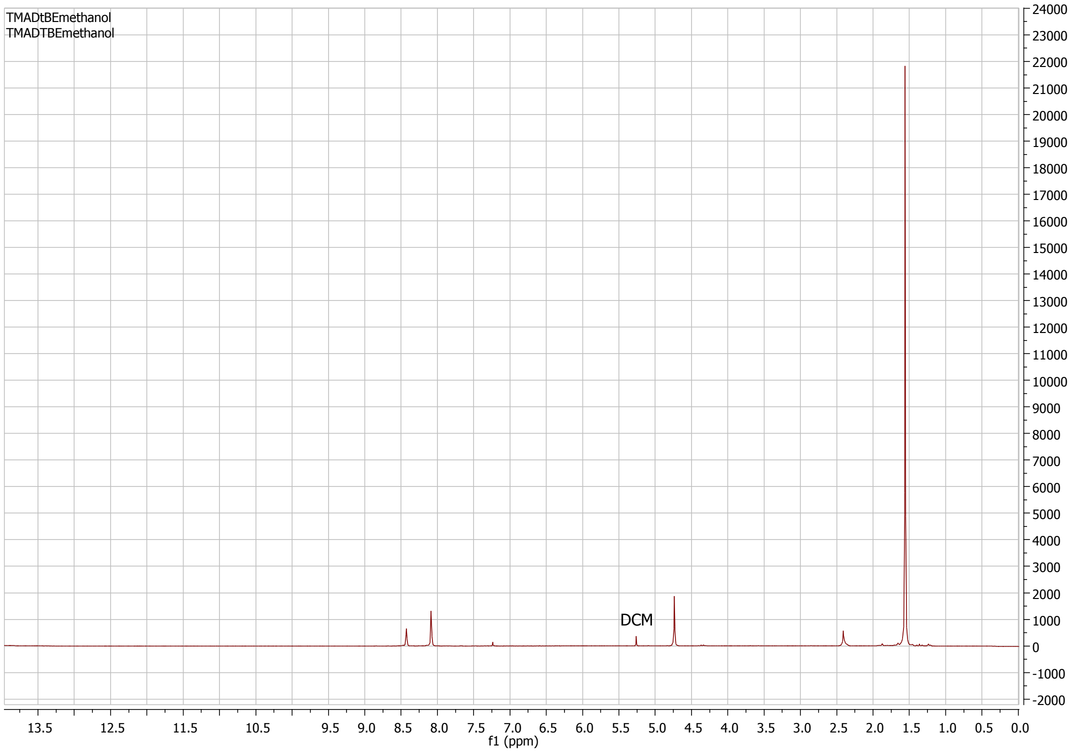

^{13}C NMR

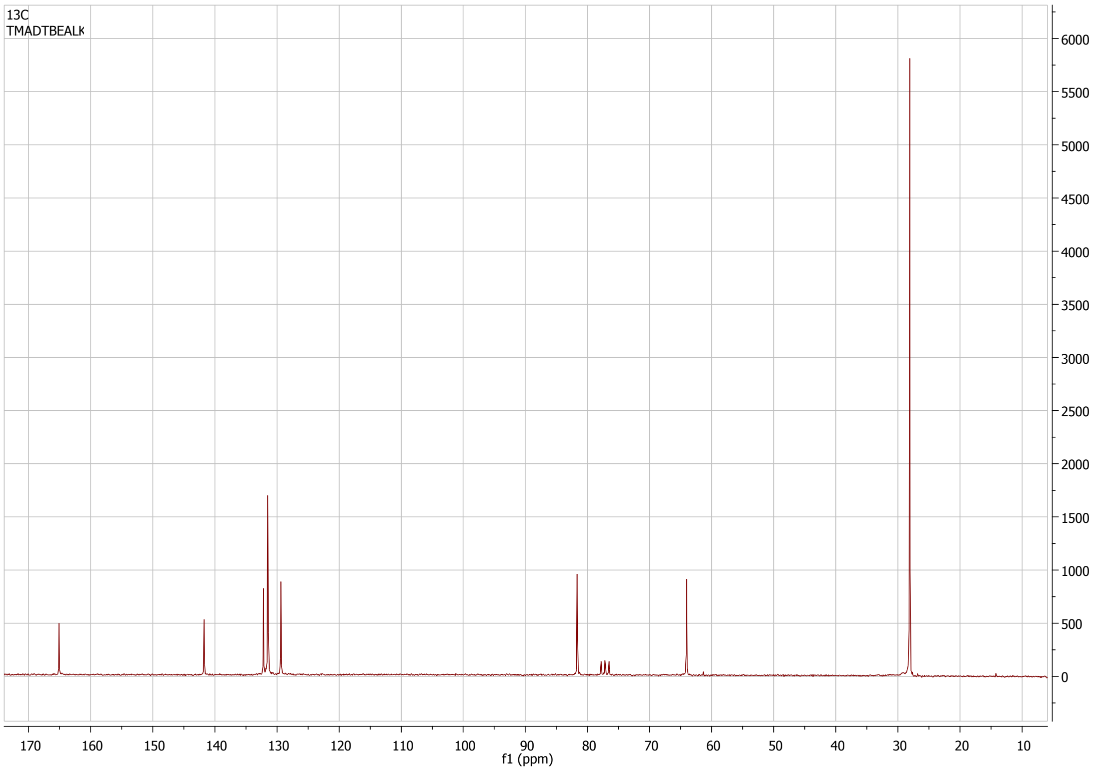

Mass Spectrum

Elemental Composition Report **Page 1**

Single Mass Analysis
Tolerance = 5.0 PPM / DBE: min = -1.5, max = 70.0
Element prediction: Off
Number of isotope peaks used for i-FIT = 9

Monoisotopic Mass, Odd and Even Electron Ions
18 formula(e) evaluated with 1 results within limits (all results (up to 1000) for each mass)
Elements Used:
C: 0-30 H: 0-25 O: 0-7
Salamo
200203_TMADTBEalk_APCInA 16 (0.177) Cm (14:20-(3:11+36:72)) TOF MS AP- 1.31e+005

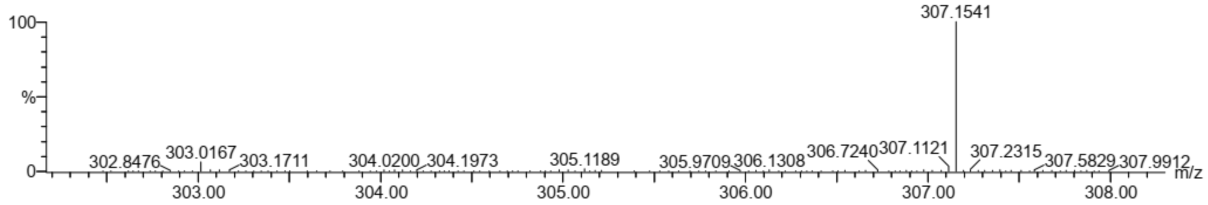

Minimum:				-1.5				
Maximum:		5.0	5.0	70.0				
Mass	Calc. Mass	mDa	PPM	DBE	i-FIT	Norm	Conf(%)	Formula
307.1541	307.1545	-0.4	-1.3	6.5	271.9	n/a	n/a	C17 H23 O5

Spectra of the dendrimer **4**

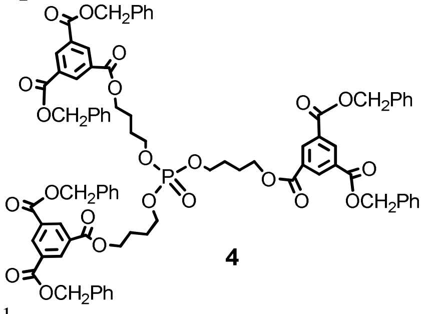

4

^{1}H NMR

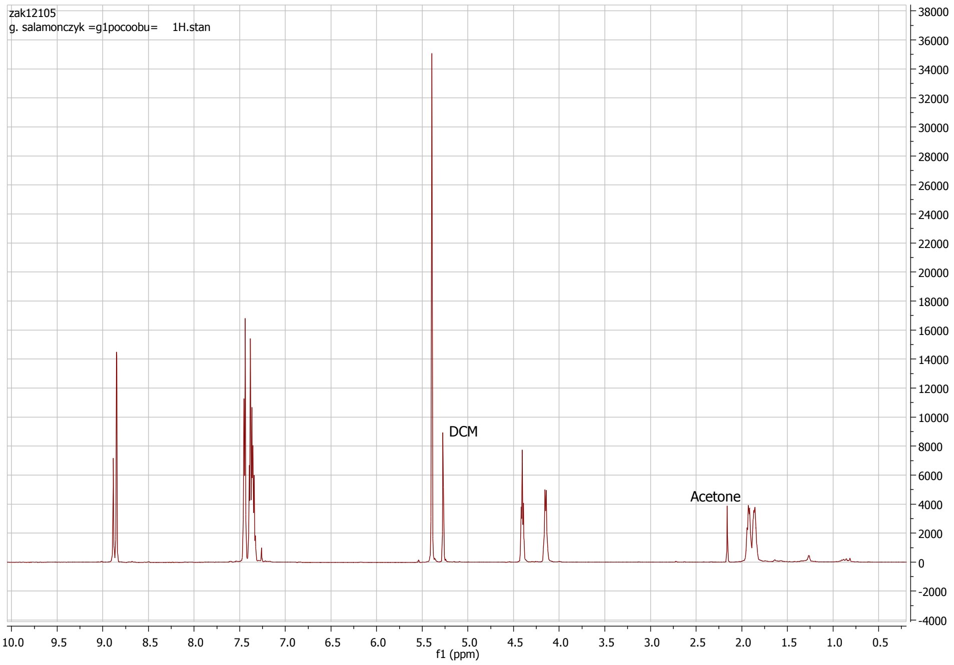

^{13}C NMR

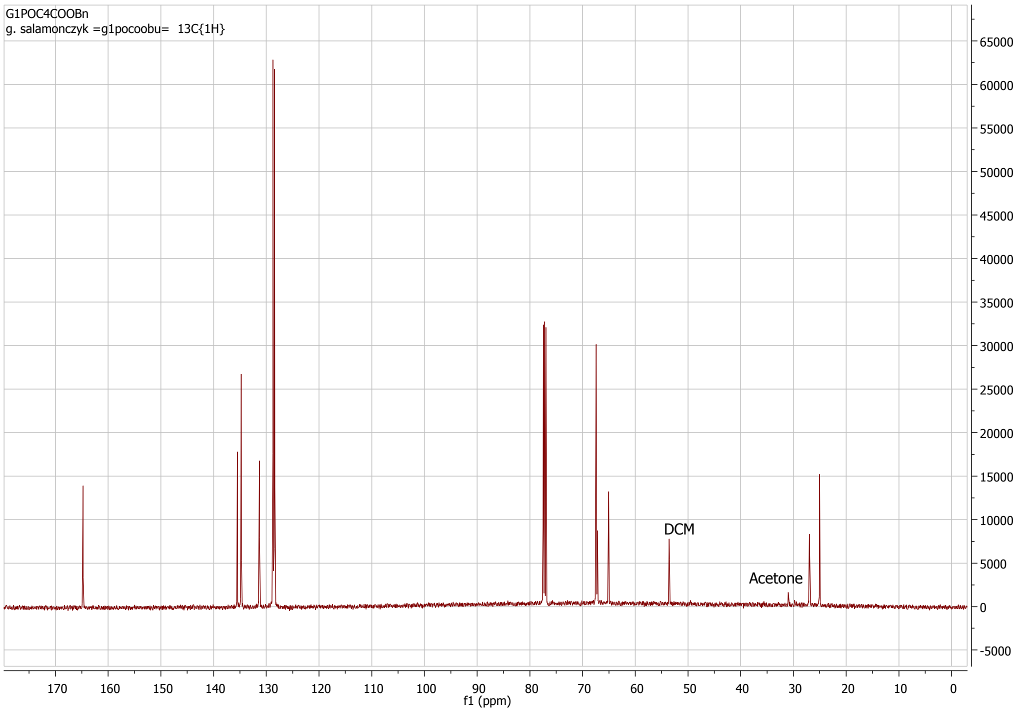

$^{13}C\{^{31}P\}$ NMR

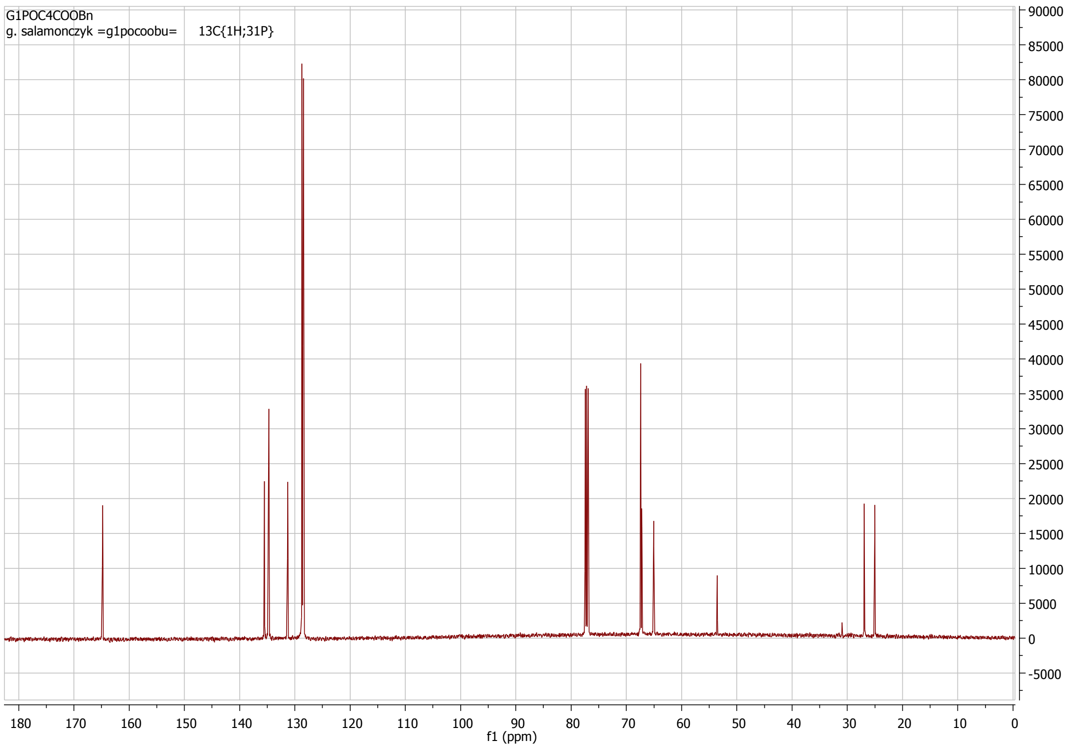

^{13}C DEPT-135 NMR

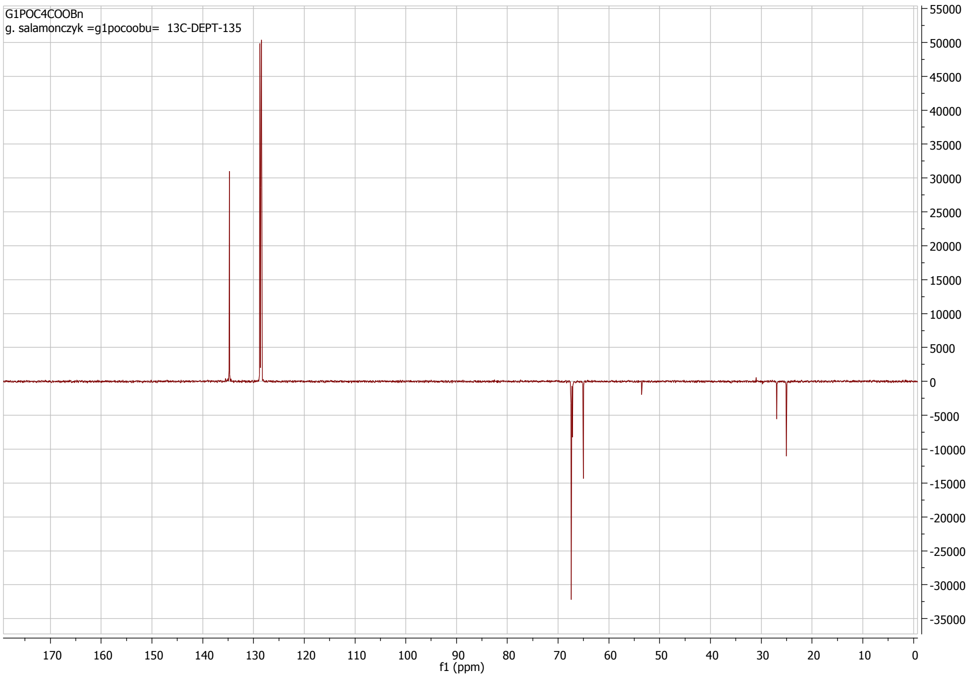

^{31}P NMR

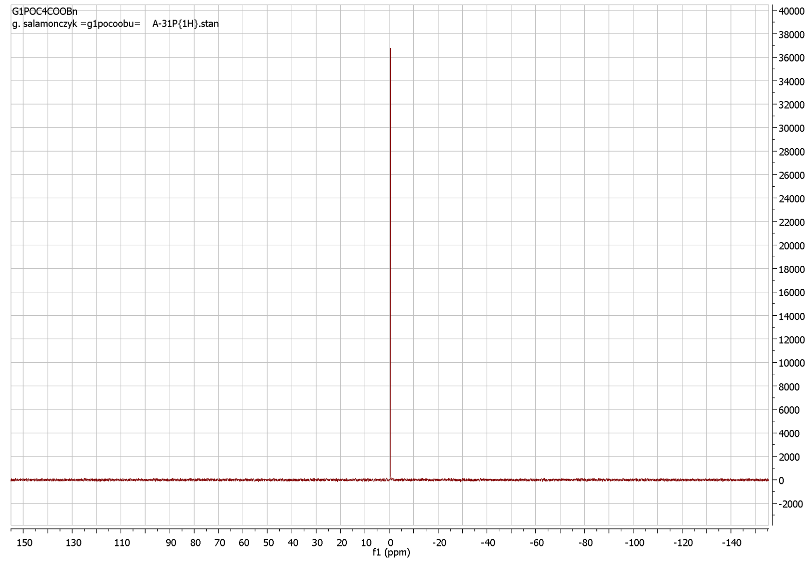

Mass Spectrum

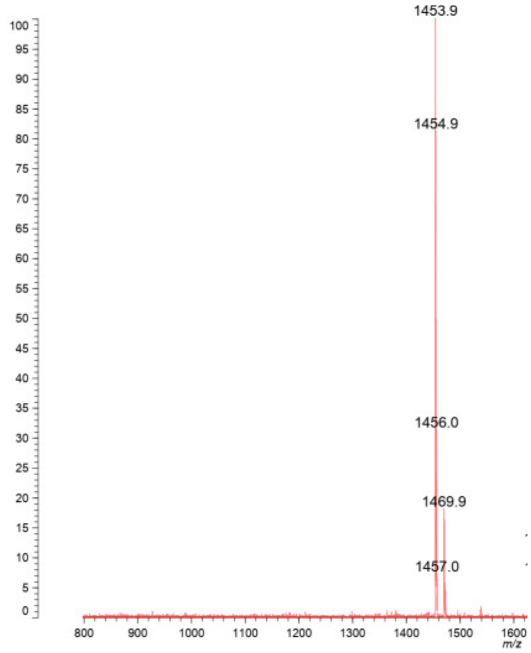

Spectra of the dendrimer **5** (**6**)

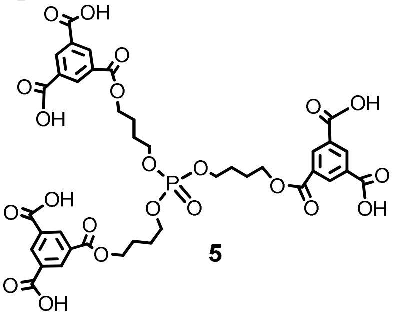

5

^{1}H {^{31}P} NMR

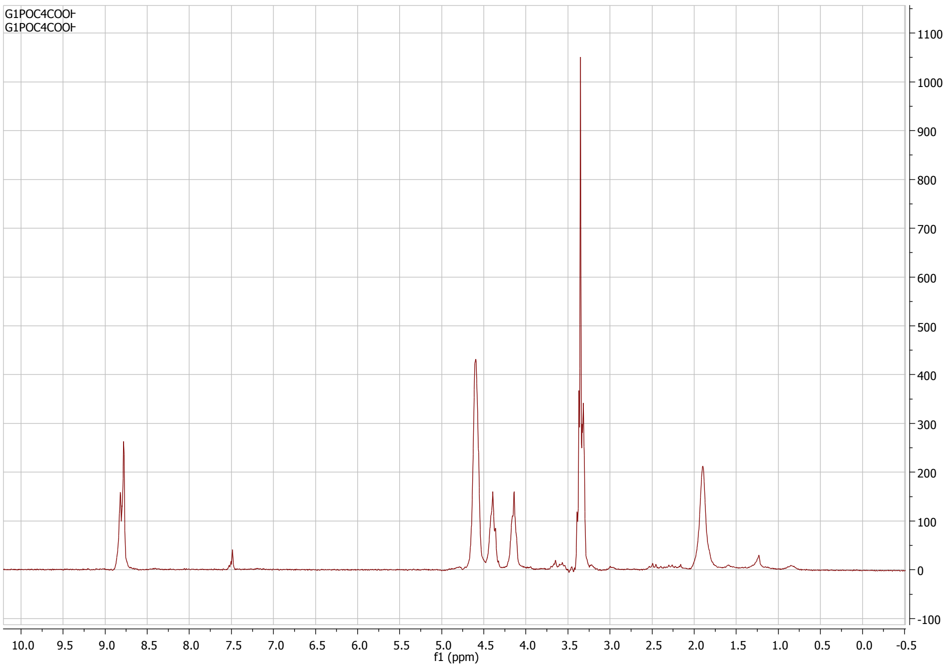

^{13}C NMR

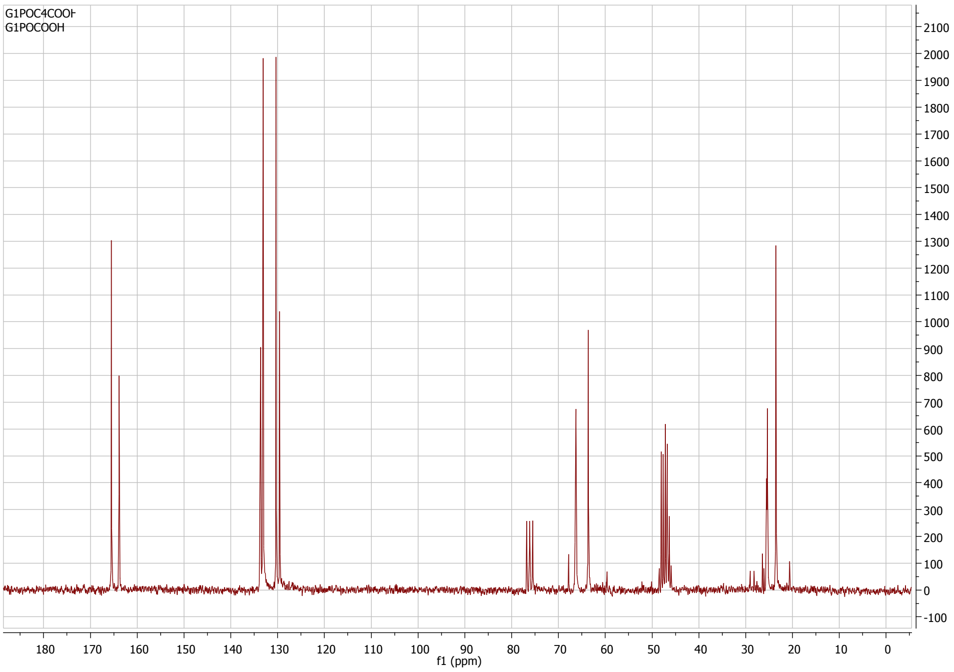

^{31}P NMR

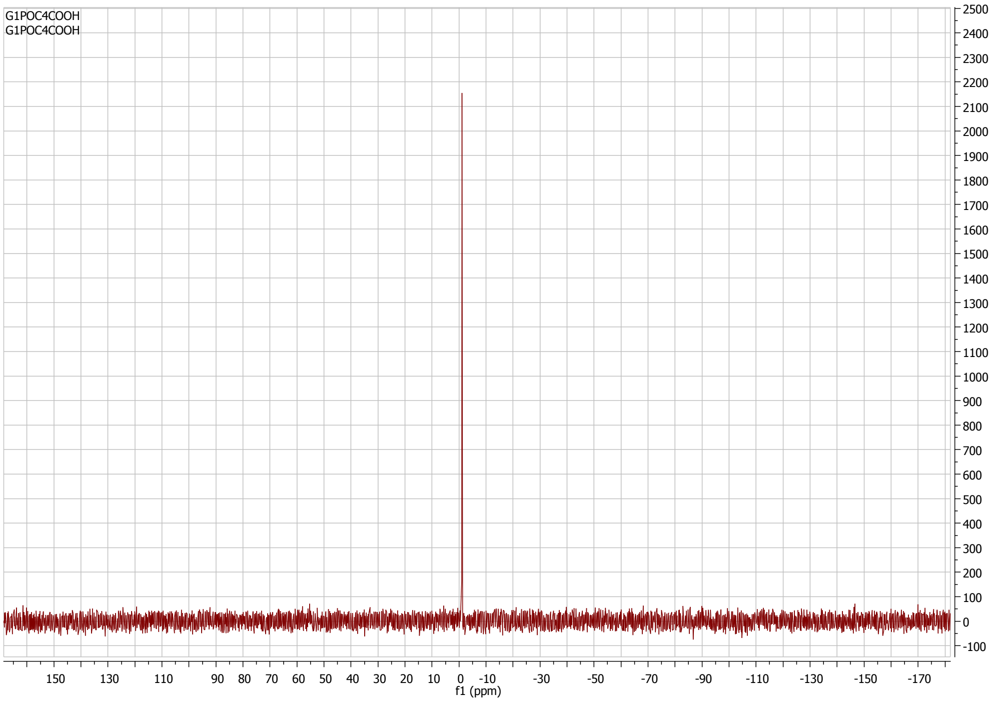

Mass Spectrum

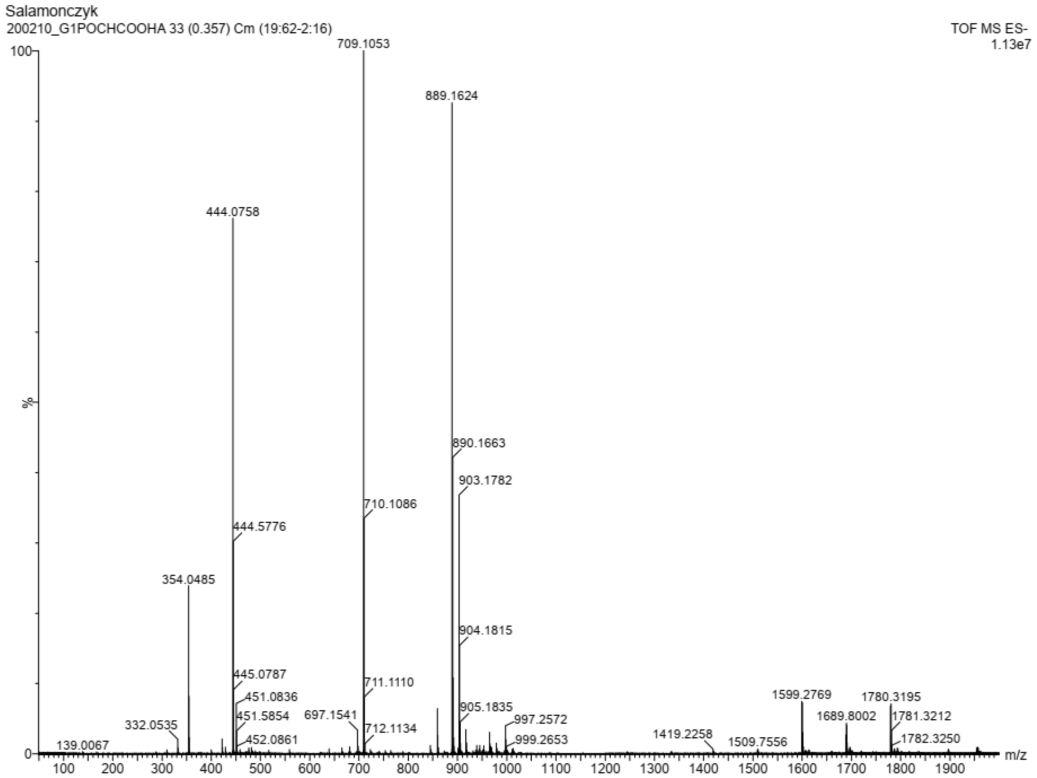

Spectra of the dendrimer **7**

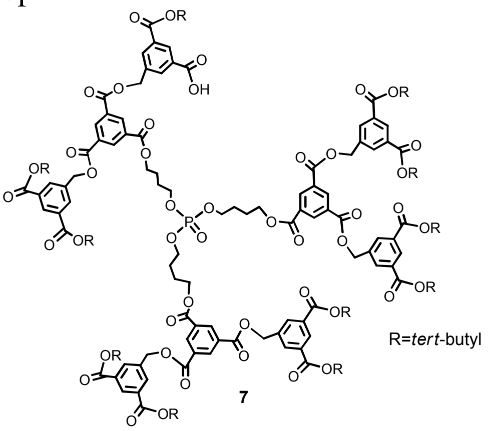

^{1}H NMR

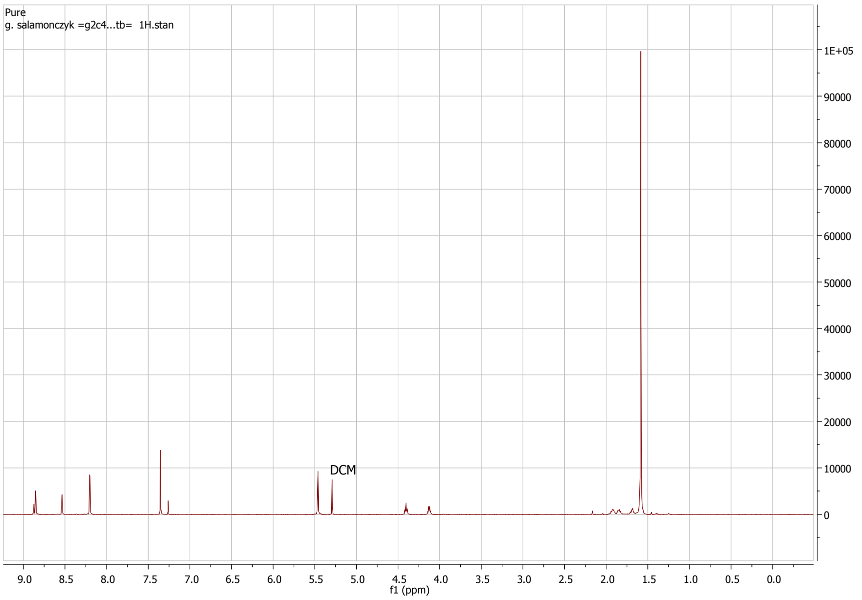

^{1}H {^{31}P} NMR

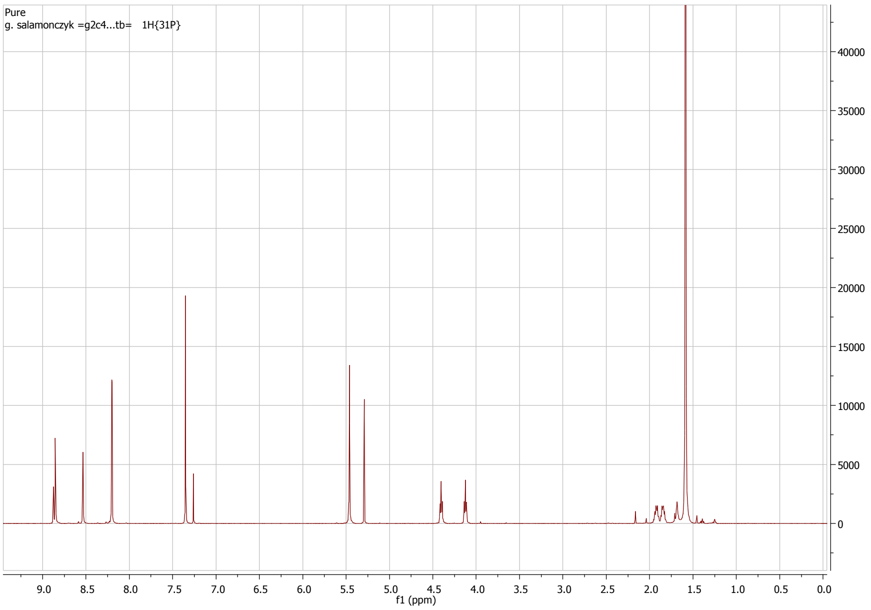

^{13}C NMR

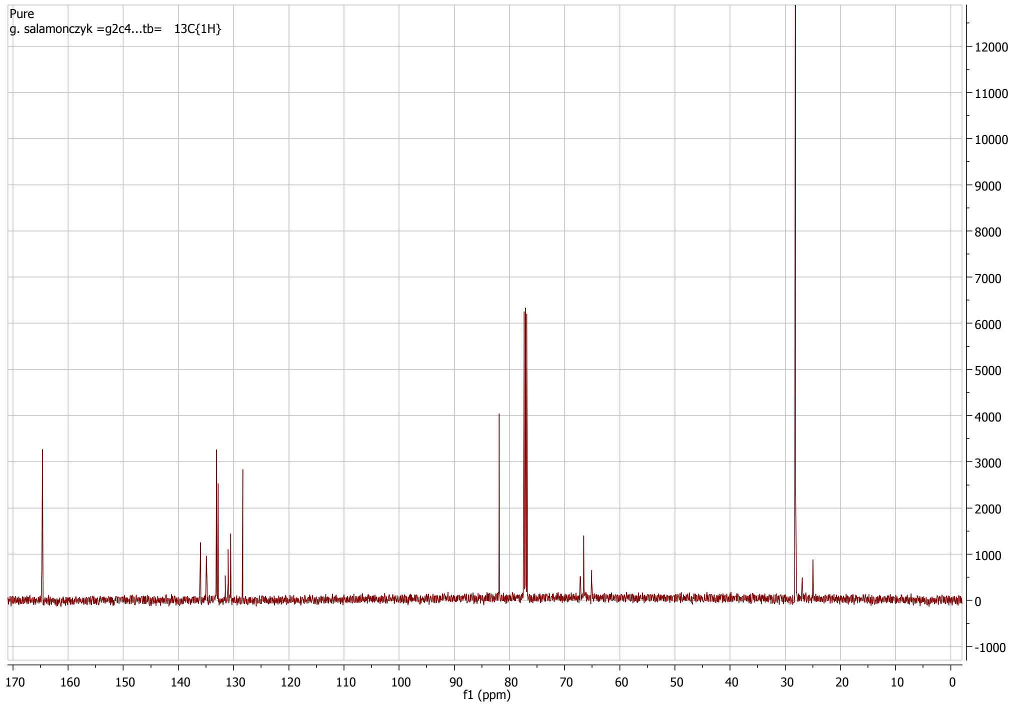

^{13}C DEPT-135 NMR

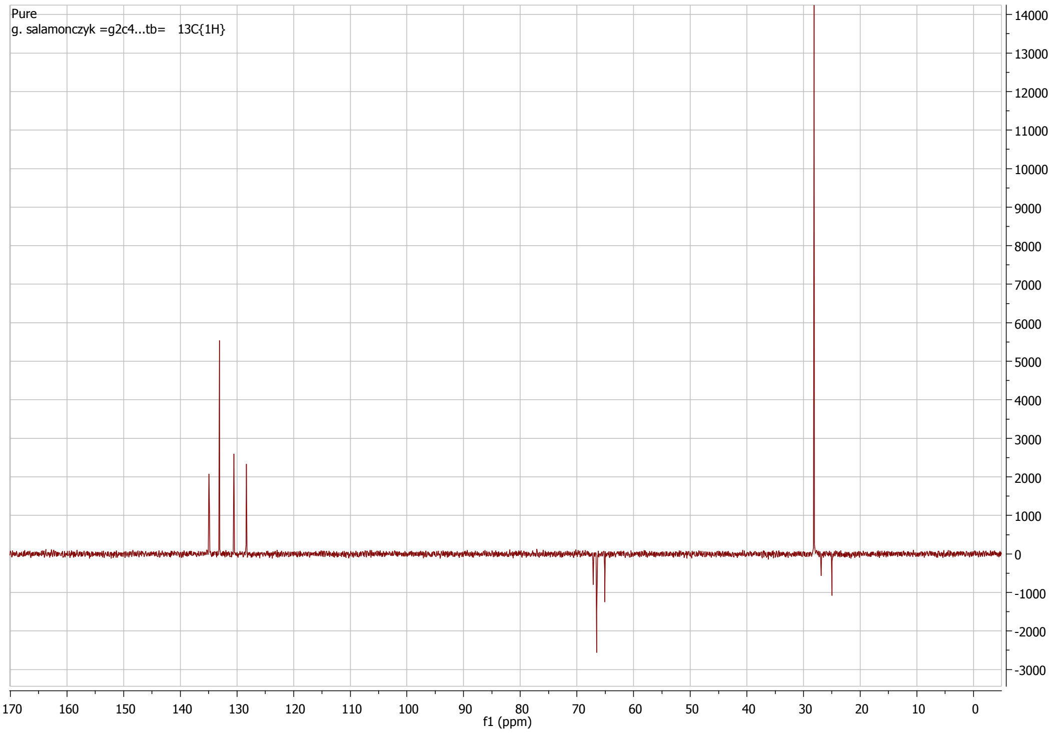

^{31}P NMR

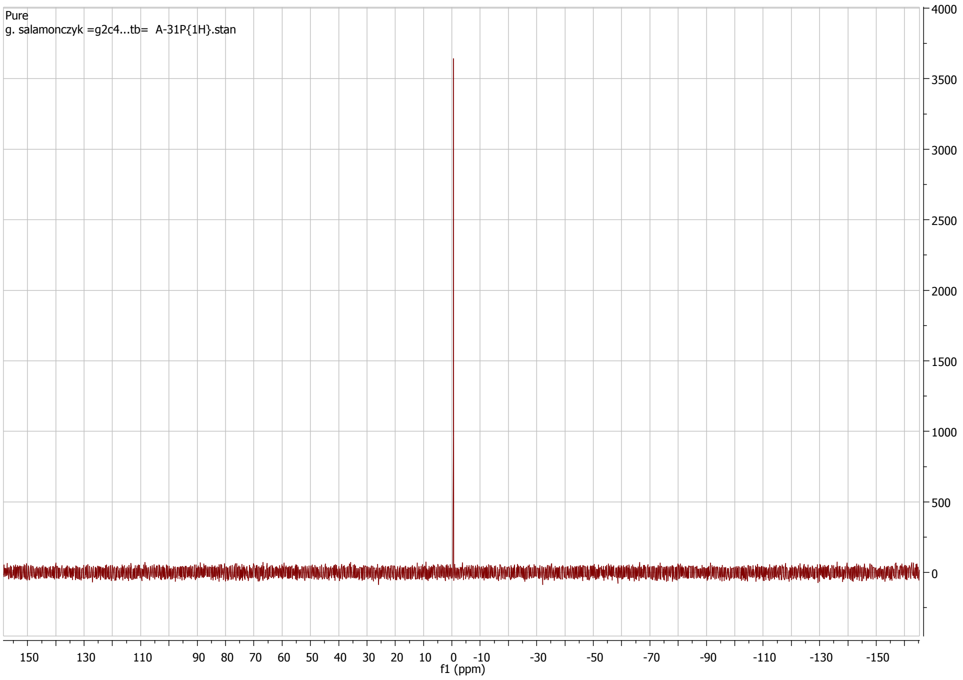

Mass Spectrum

G.Salamonczyk, G2C4POCOCOOTB; linear pos

Data: hr870001.F8[c] 10 Jul 2020 13:01 Cal: PEG 2000 (06.2020) 22 Jun 2020 12:42
Shimadzu Biotech Axima Performance 2.9.1.20100121: Mode Linear_2018, Power: 80, Blanked, P.Ext. @ 3000 (bin 78)
%Int. 1.3 mV[sum= 266 mV] Profiles 1-200 Smooth Av 5 -Baseline 20

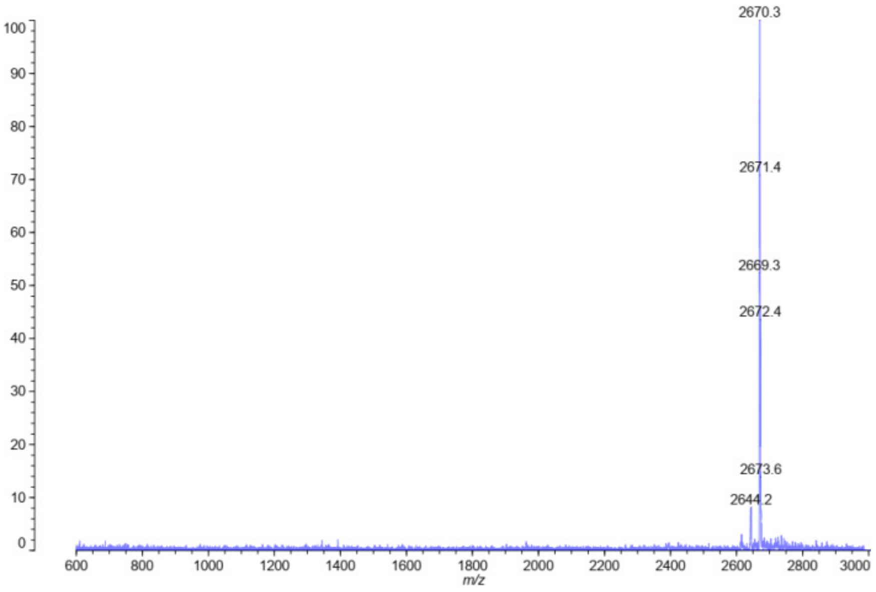

Spectra of the dendrimer **8** (**9**)

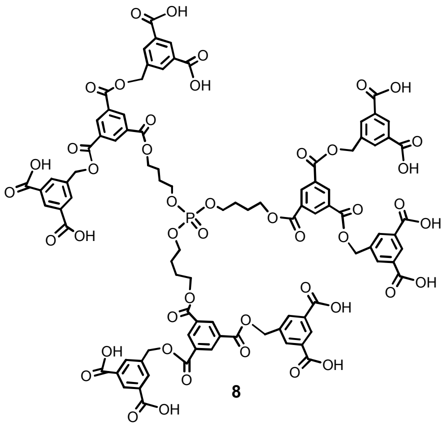

8

^{1}H NMR

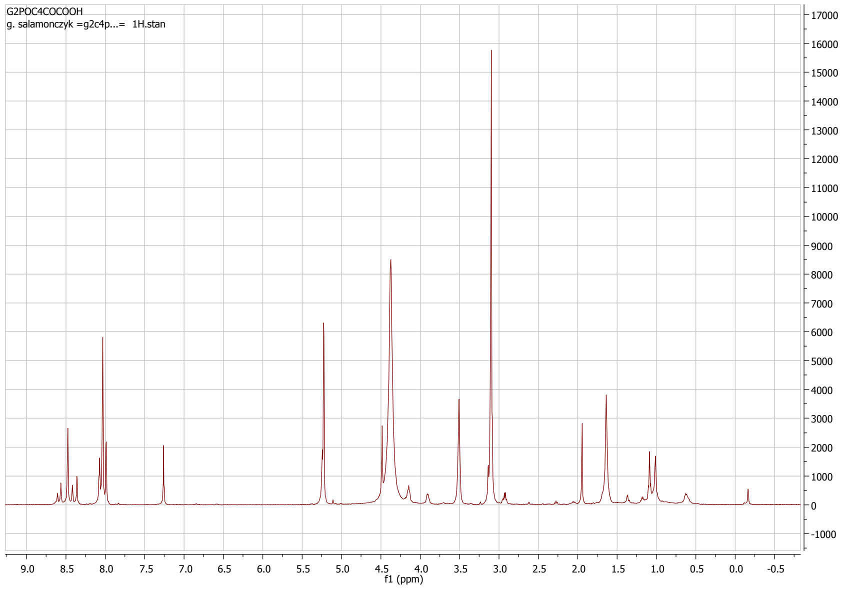

^{1}H {^{31}P} NMR

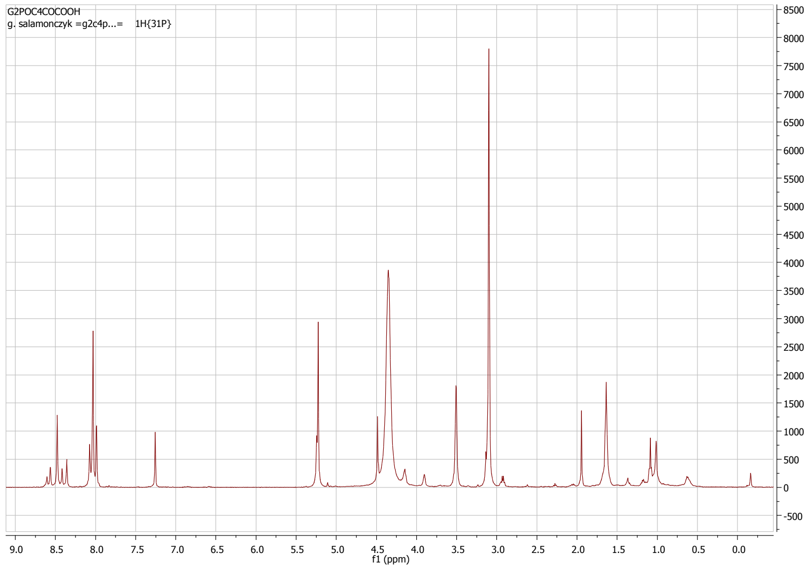

^{31}P NMR

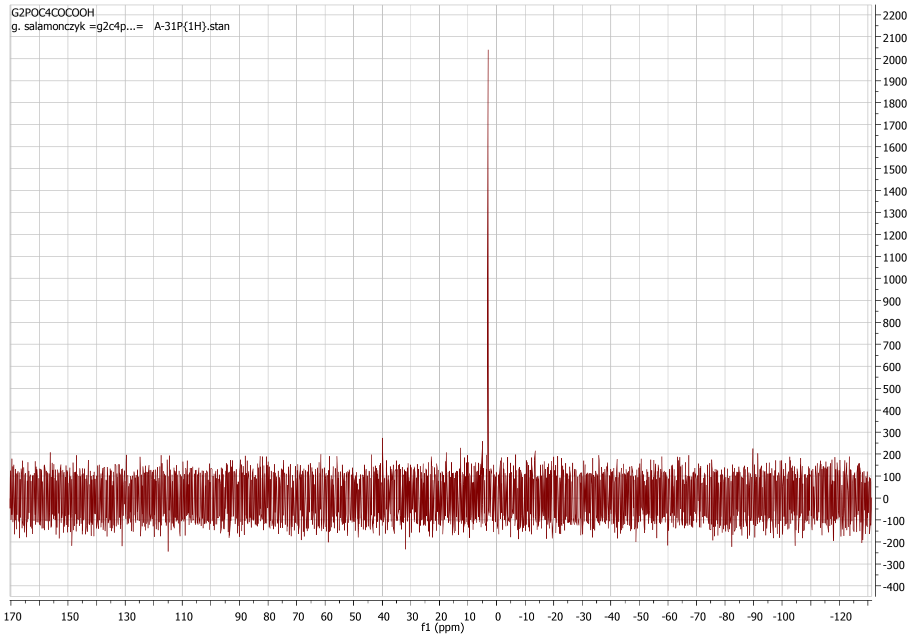

Mass Spectrum

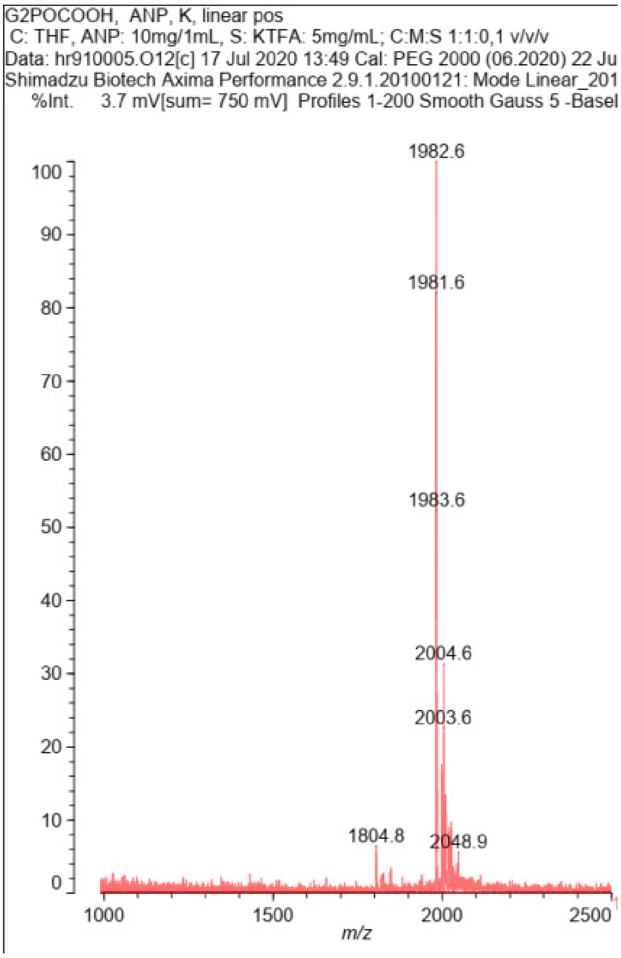

Spectra of the dendrimer **10**

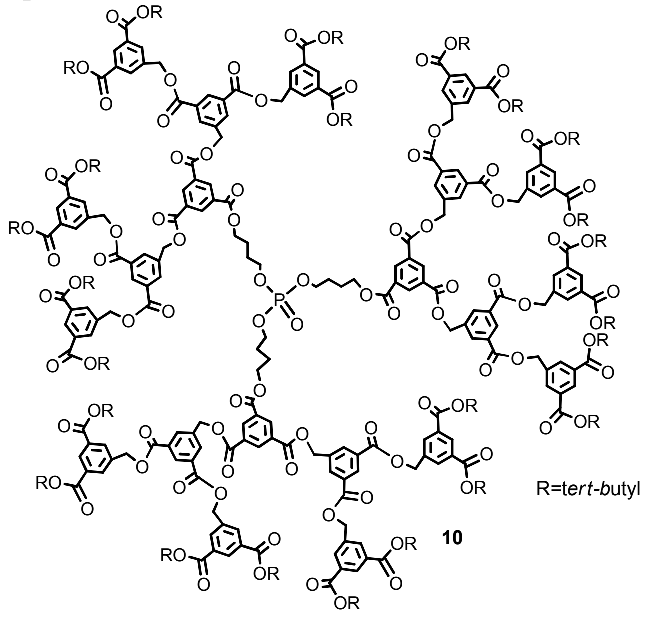

^{1}H NMR

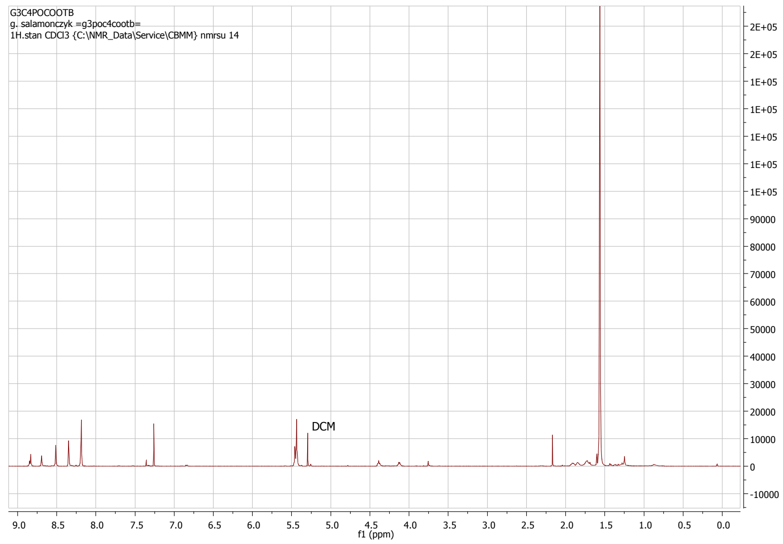

^{1}H NMR – aromatic range expansion

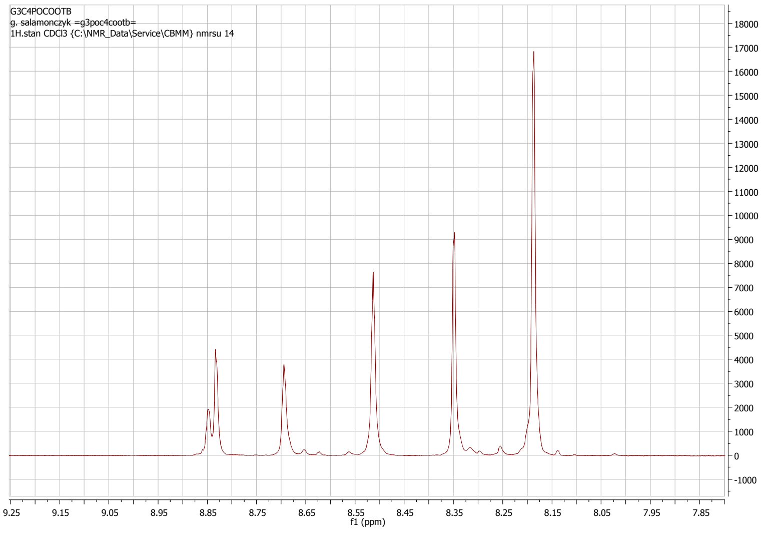

^{13}C NMR

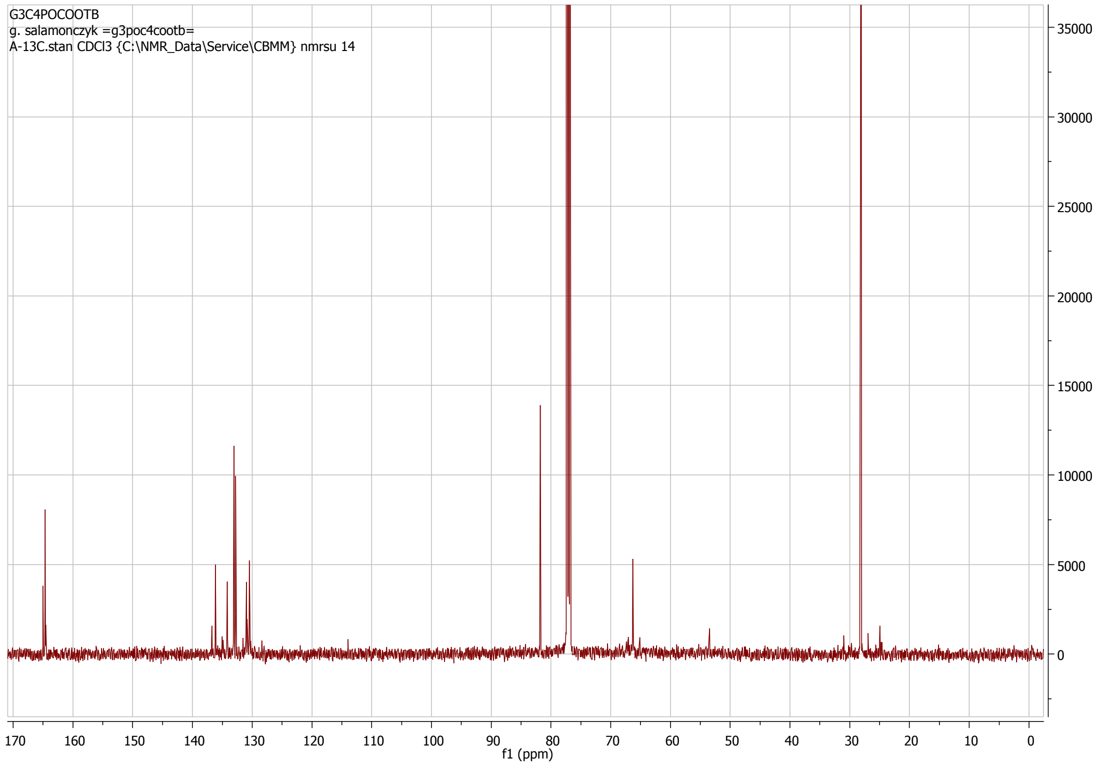

^{13}C NMR – aromatic and carbonyl range expansion

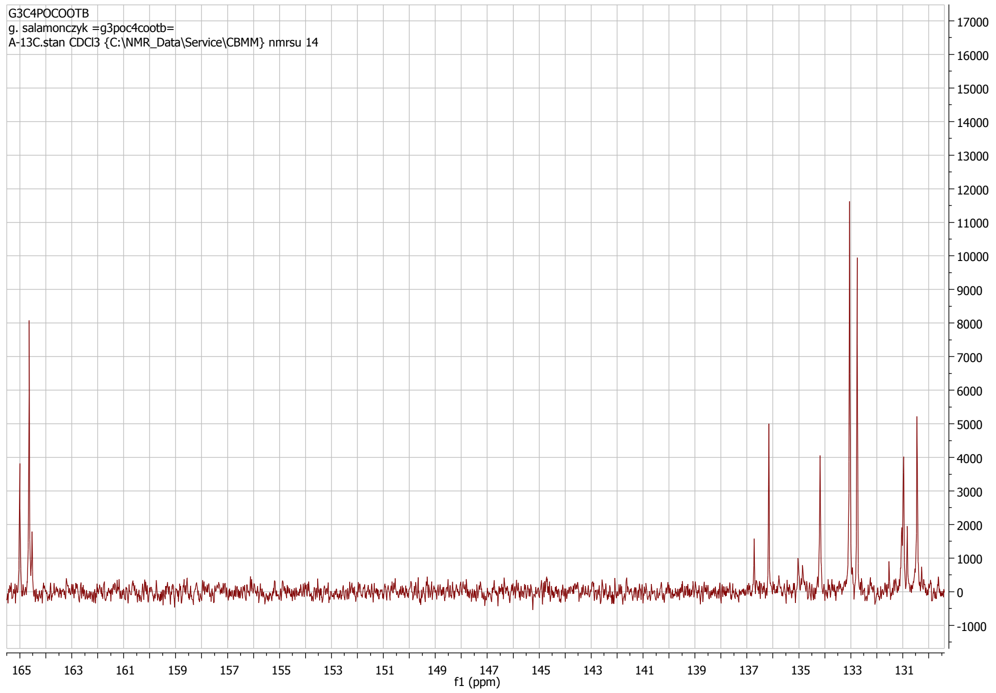

^{13}C NMR – DEPT 135

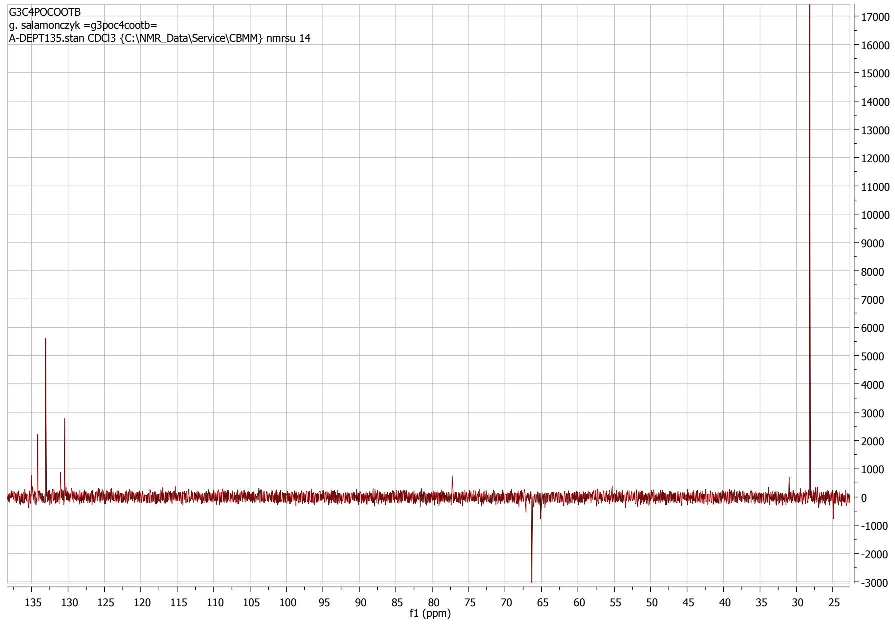

^{31}P NMR

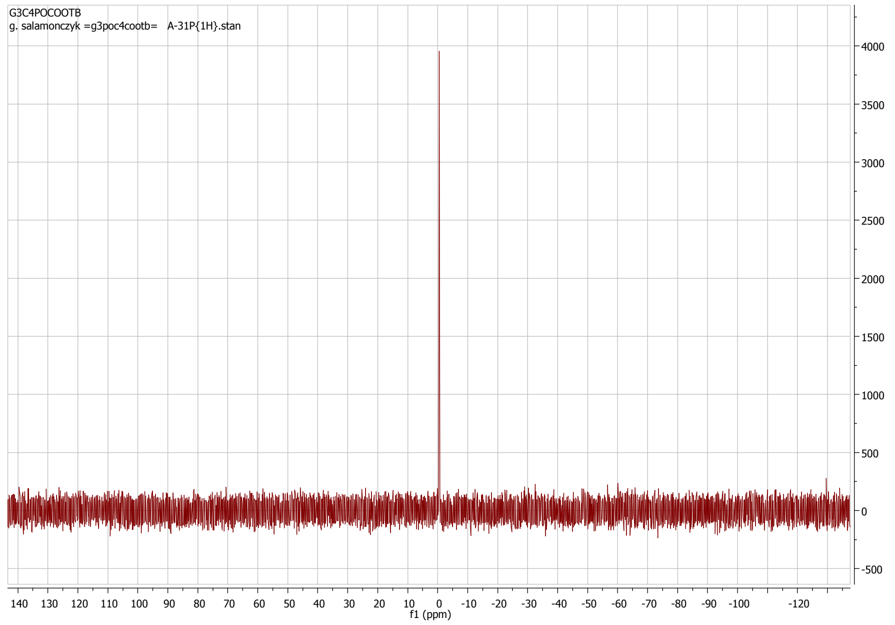

MALDI TOF Mass Spectrum

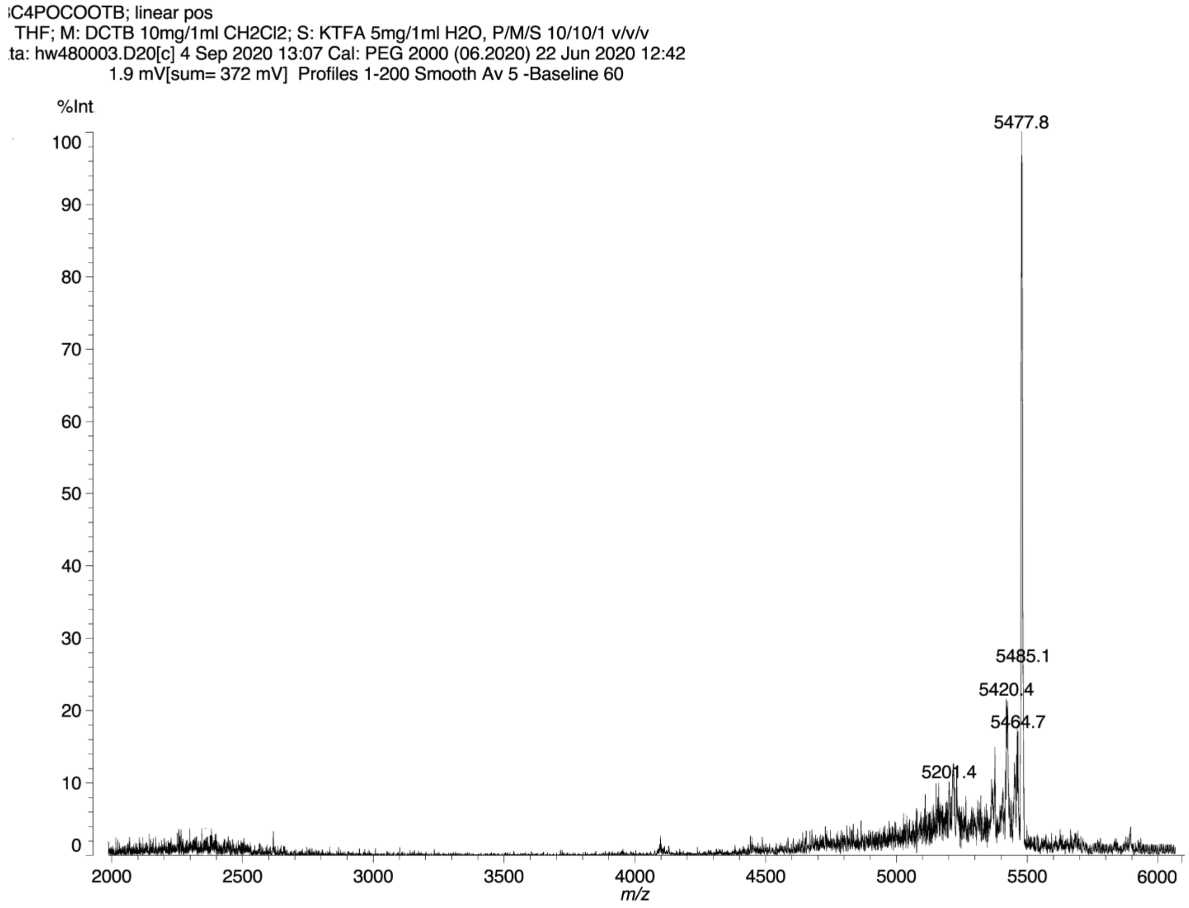

Spectra of the dendrimer **11** (**12**)

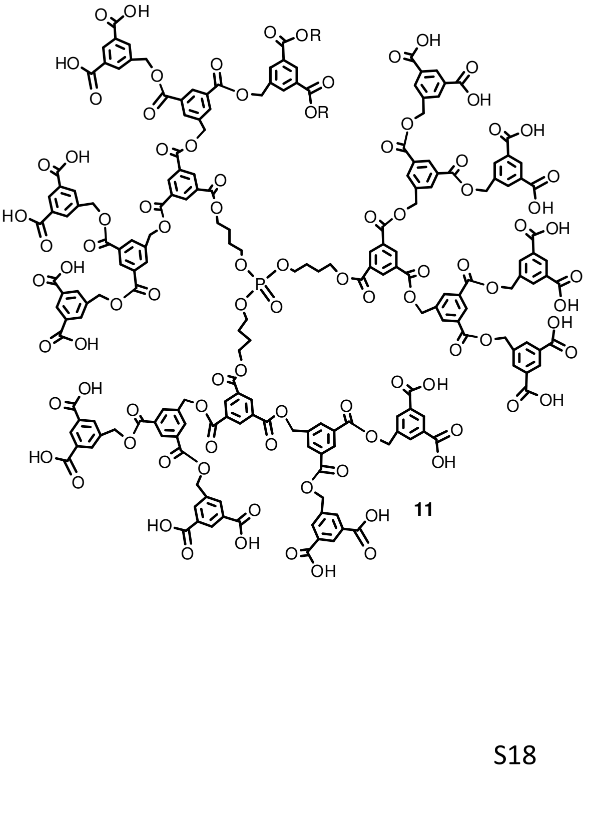

11

^{1}H NMR

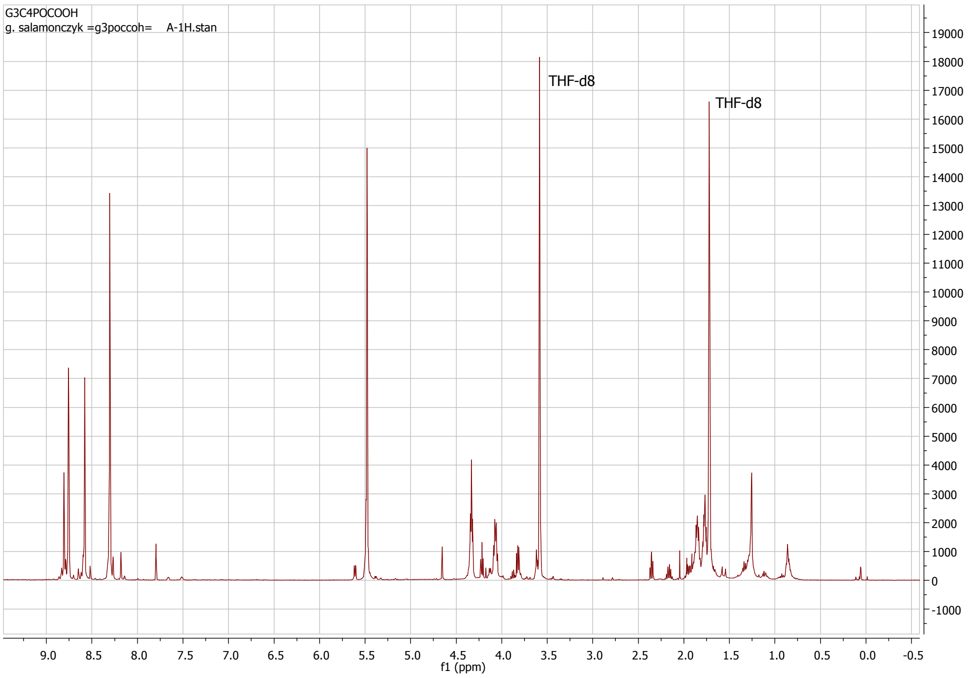

$^{1}H\{^{31}P\}$ NMR

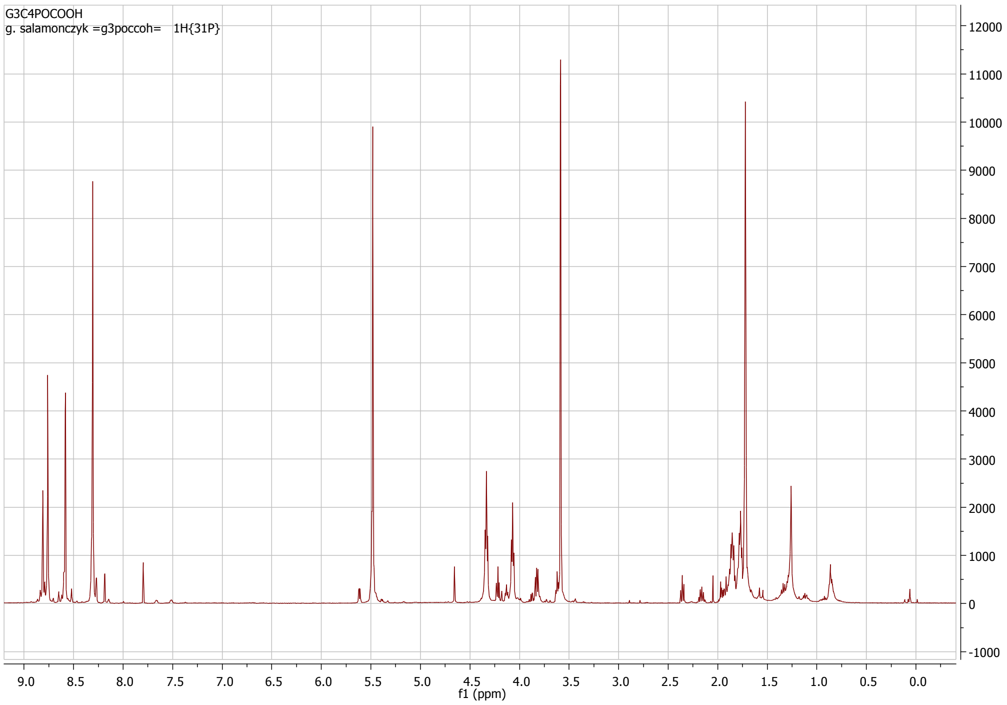

^{13}C NMR

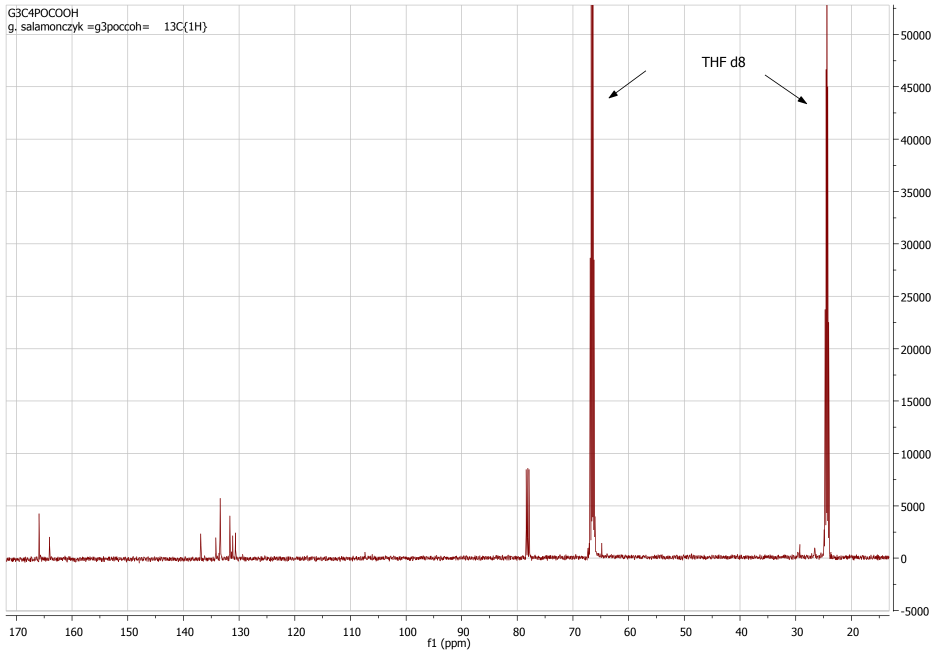

^{13}C NMR – aromatic and carbonyl range expansion

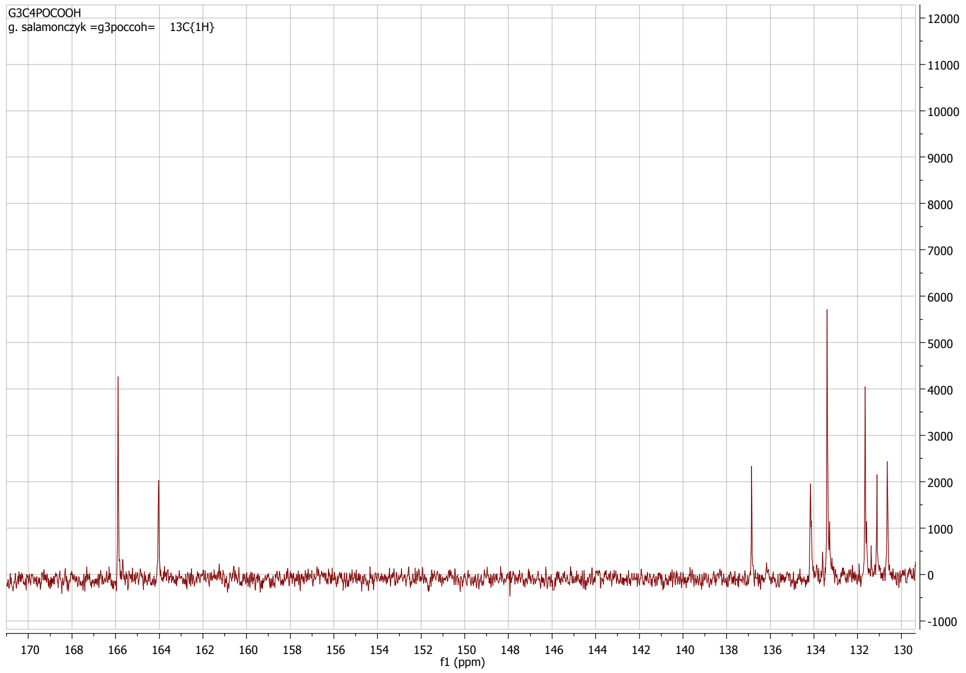

^{31}P NMR

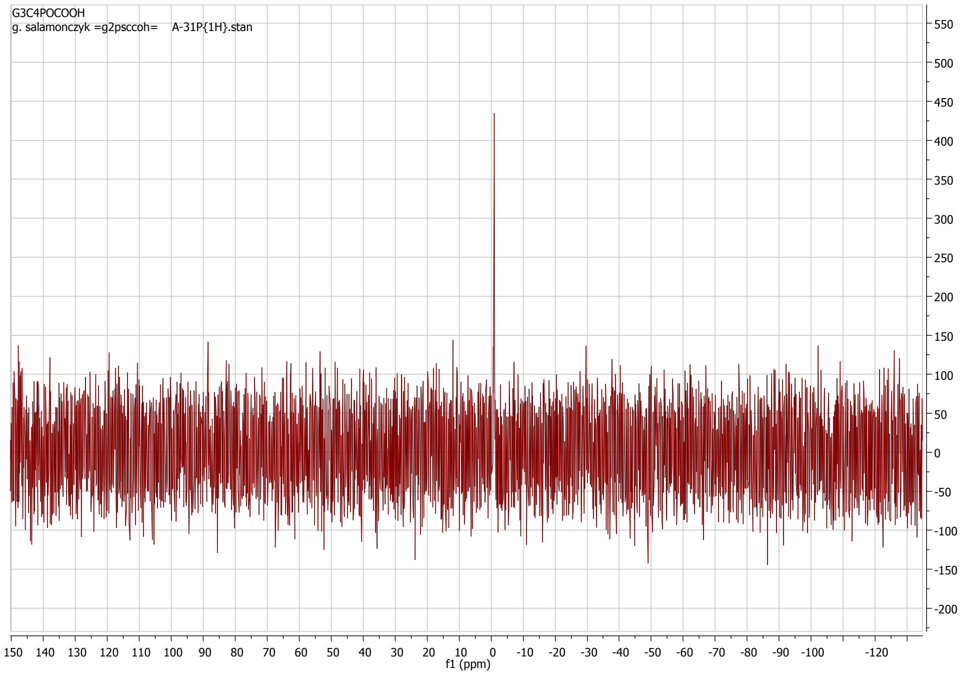

Spectra of the dendrimer **14**

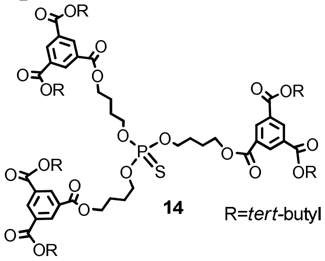

^{1}H NMR

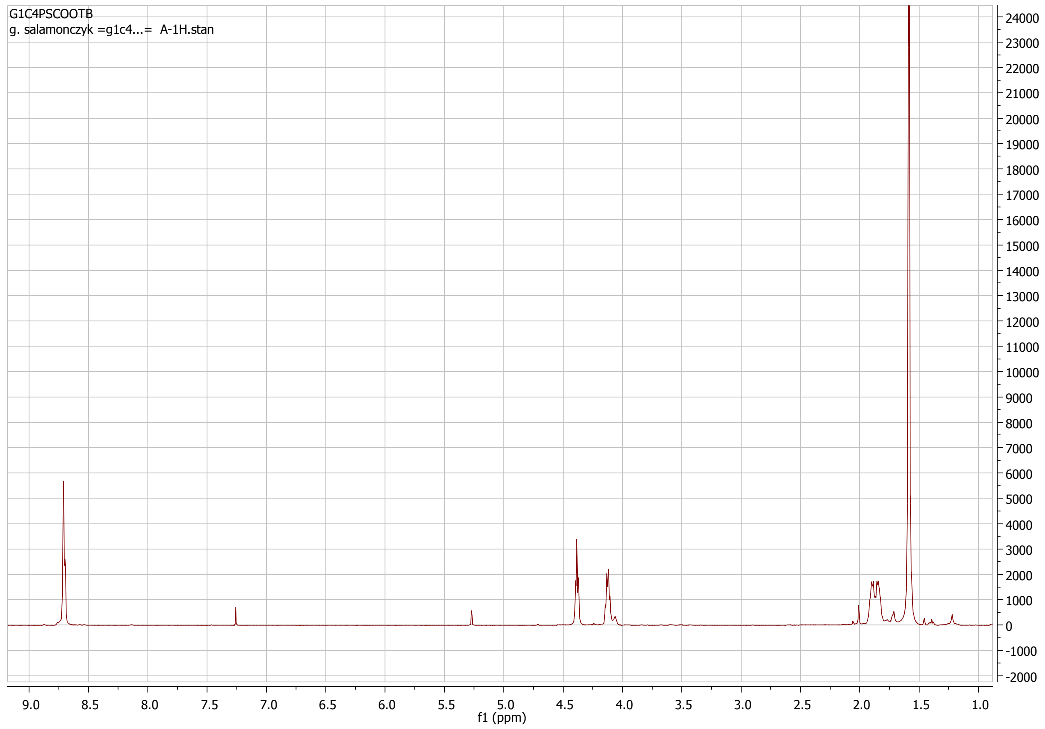

$^{1}H\ \{^{31}P\}$ NMR

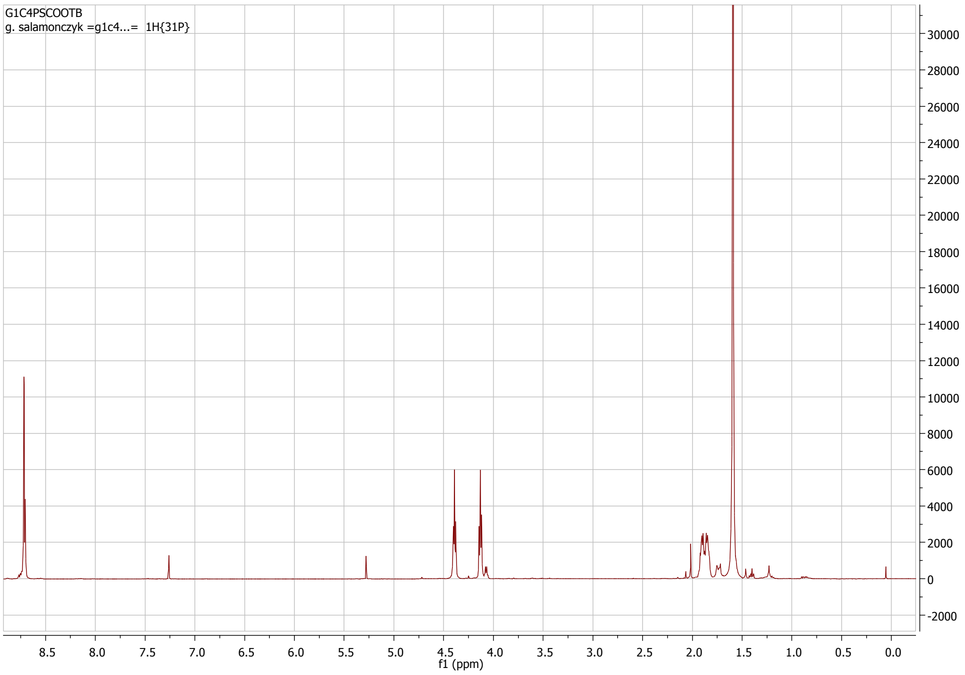

^{13}C NMR

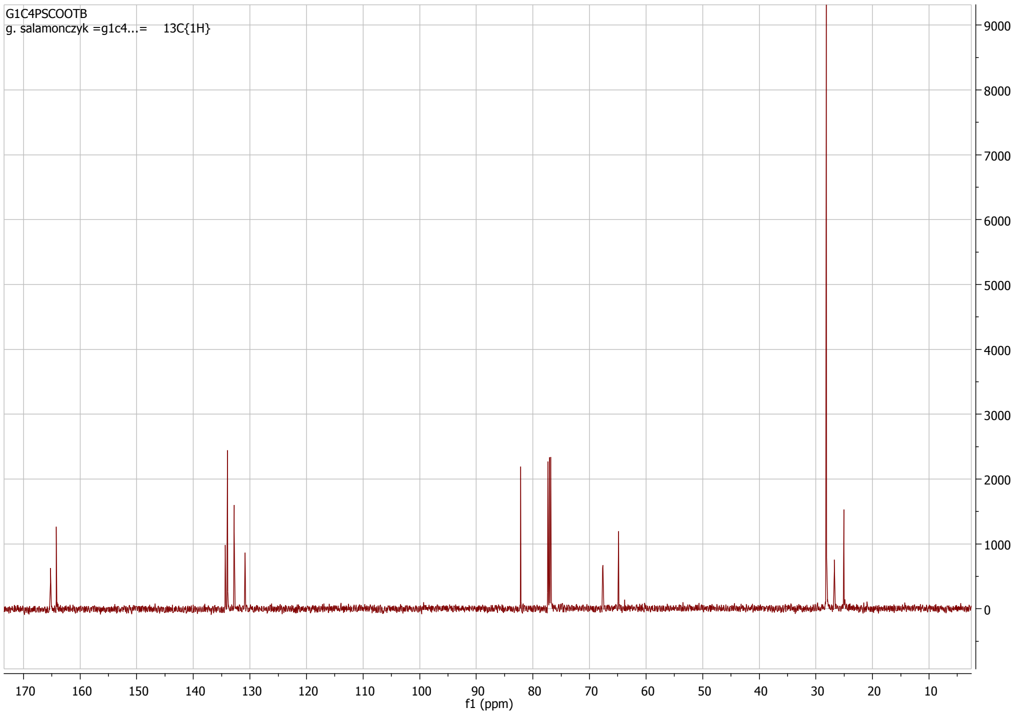

^{13}C NMR – DEPT 135

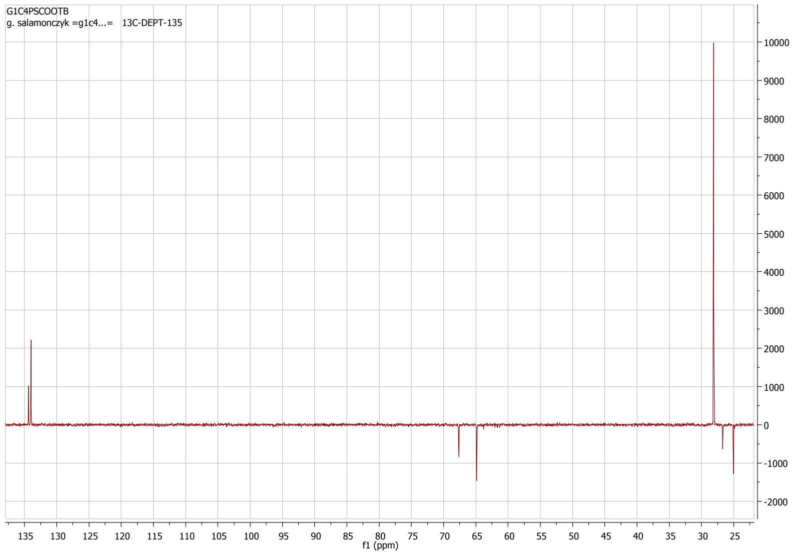

^{31}P NMR

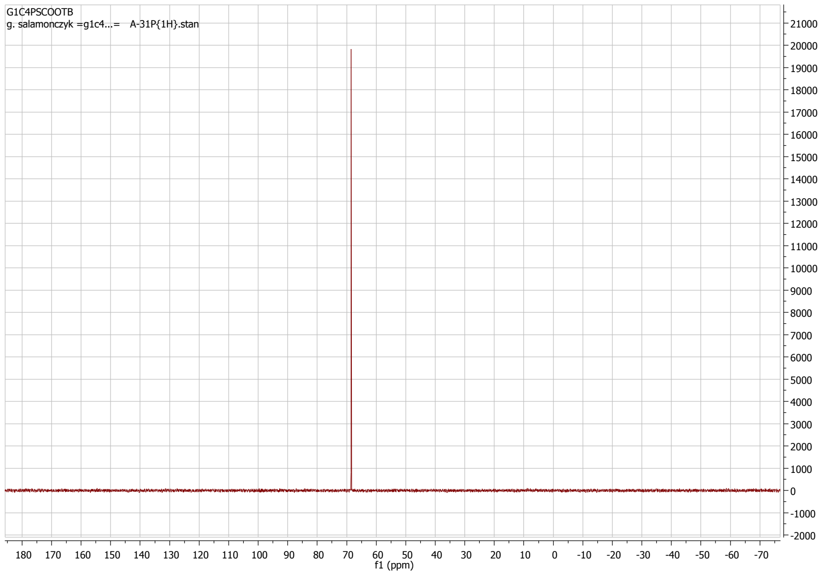

Mass Spectrum

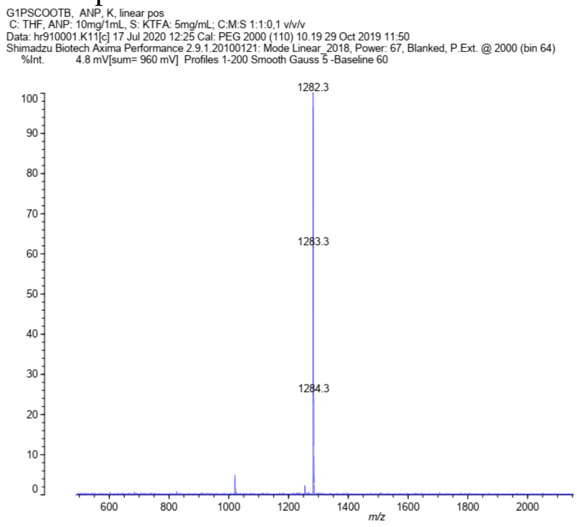

Spectra of the dendrimer **15** (**16**)

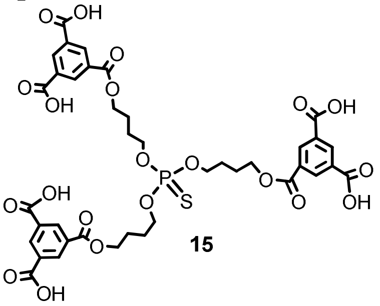

^{1}H NMR

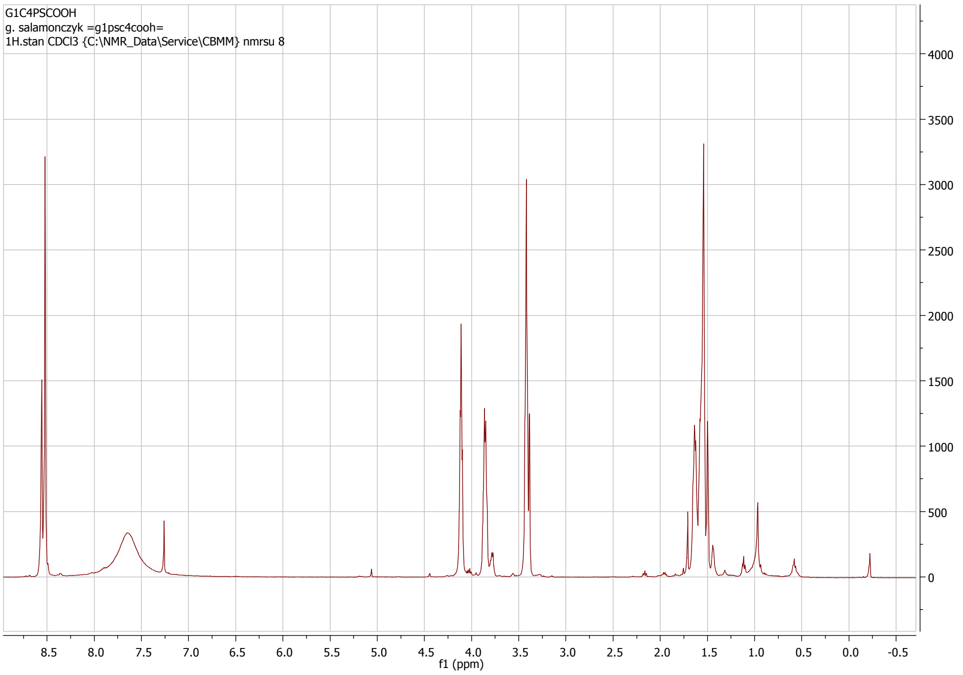

^{13}C NMR

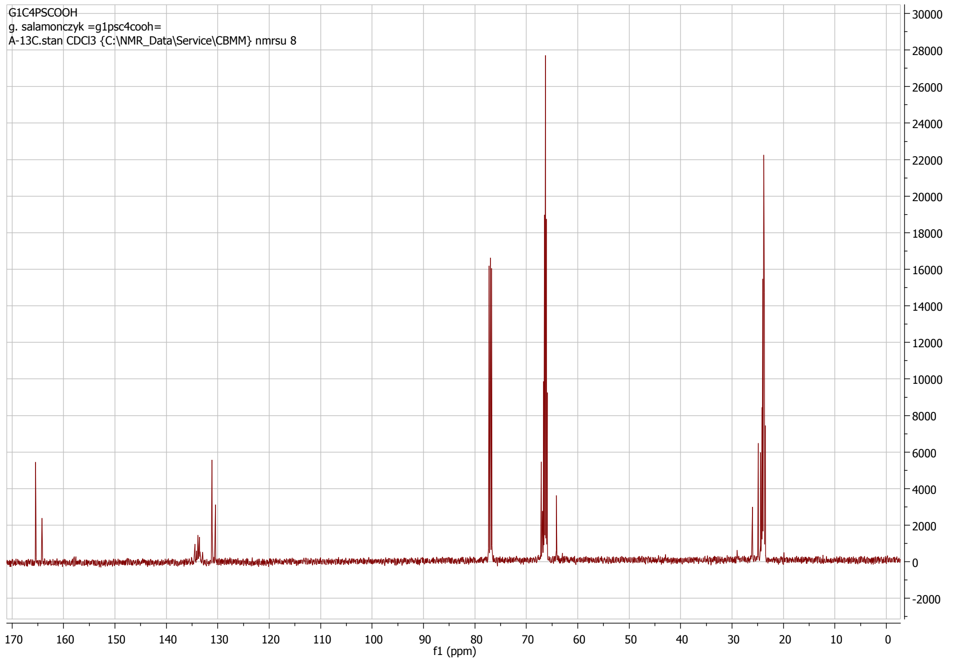

^{31}P NMR

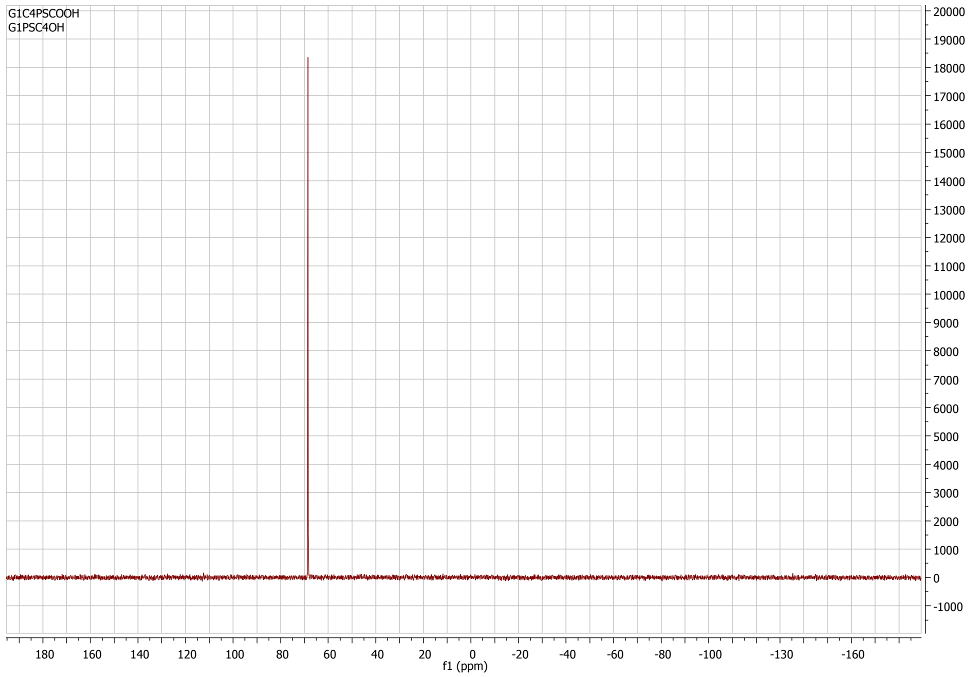

Mass Spectrum

Elemental Composition Report Page 1

Single Mass Analysis
Tolerance = 5.0 PPM / DBE: min = -1.5, max = 70.0
Element prediction: Off
Number of isotope peaks used for i-FIT = 9

Monoisotopic Mass, Odd and Even Electron Ions
409 formula(e) evaluated with 6 results within limits (all results (up to 1000) for each mass)
Elements Used:
C: 0-45 H: 0-45 O: 0-25 P: 0-2 S: 0-2
200831_G1PSC4COOHn1A 46 (0.480) Cm (46:72-2:38)

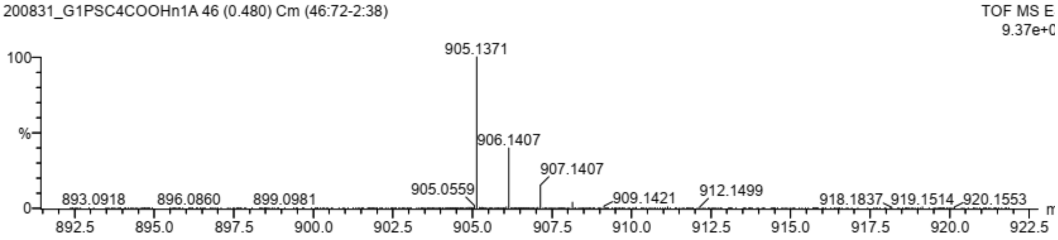

Mass	Calc. Mass	mDa	PPM	DBE	i-FIT	Norm	Conf(%)	Formula
Minimum:				-1.5				
Maximum:		5.0	5.0	70.0				
905.1371	905.1364	0.7	0.8	21.5	1913.6	2.795	6.11	C39 H38 O21 P S
	905.1398	-2.7	-3.0	16.5	1920.2	9.318	0.01	C36 H42 O21 P S2
	905.1340	3.1	3.4	12.5	1910.9	0.074	92.82	C32 H43 O24 P2 S
	905.1339	3.2	3.5	25.5	1922.3	11.459	0.00	C43 H38 O16 P S2
	905.1330	4.1	4.5	26.5	1915.9	5.071	0.63	C42 H34 O21 P
	905.1413	-4.2	-4.6	26.5	1916.3	5.452	0.43	C42 H33 O23

Spectra of the dendrimer **17**

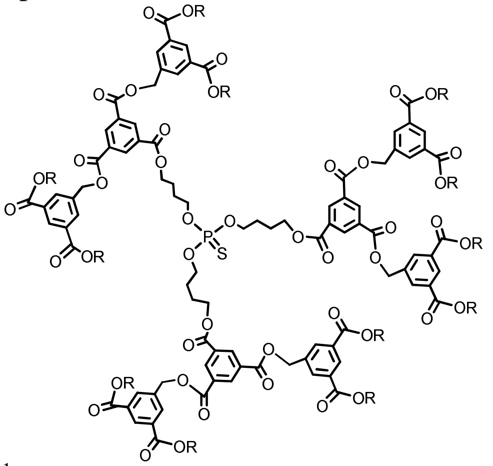

^{1}H NMR

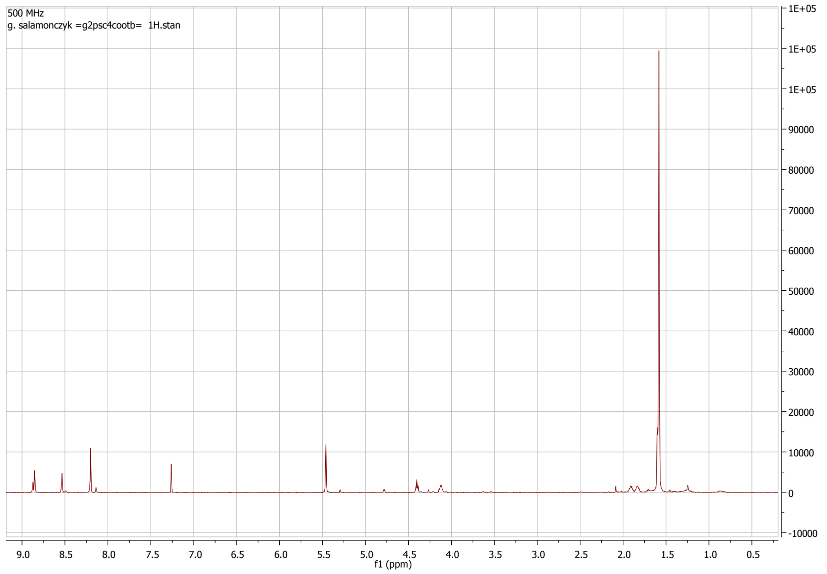

^{1}H {^{31}P} NMR

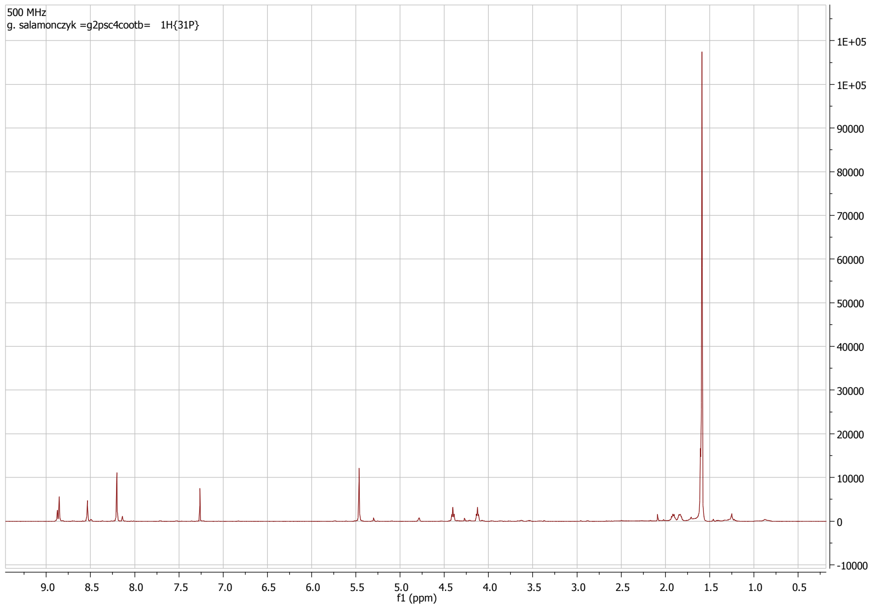

^{13}C NMR

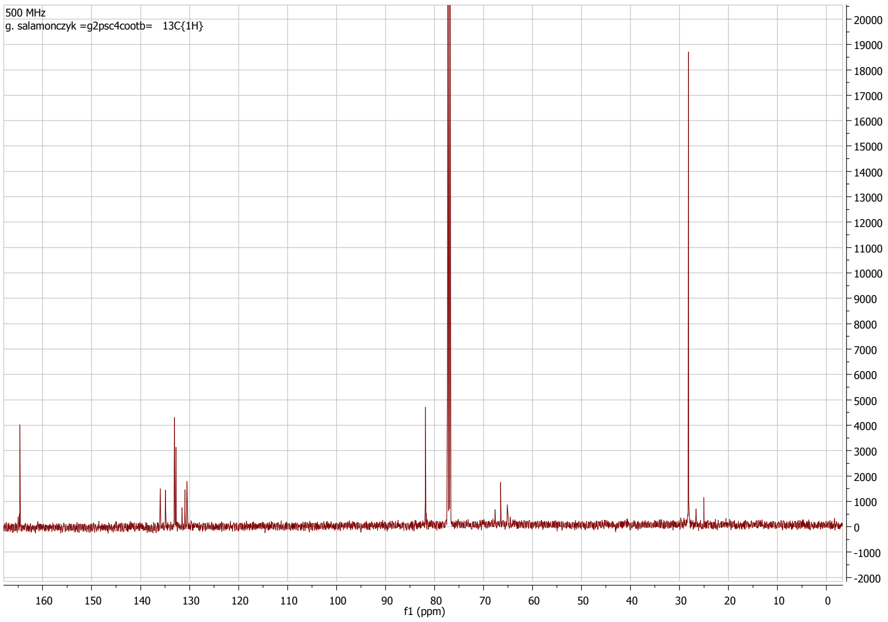

^{13}C NMR – DEPT 135

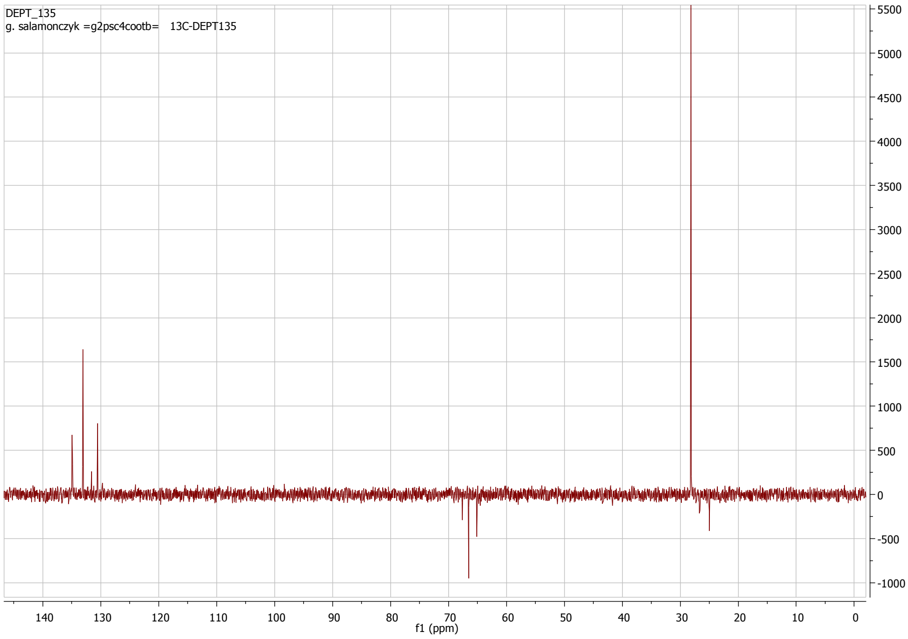

^{31}P NMR

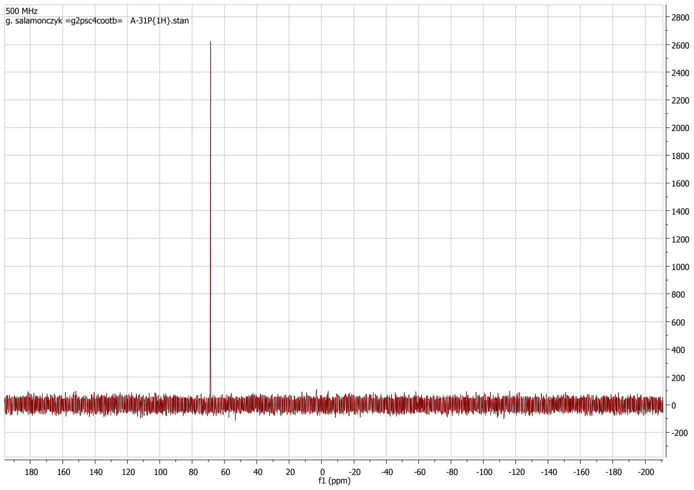

Mass Spectrum

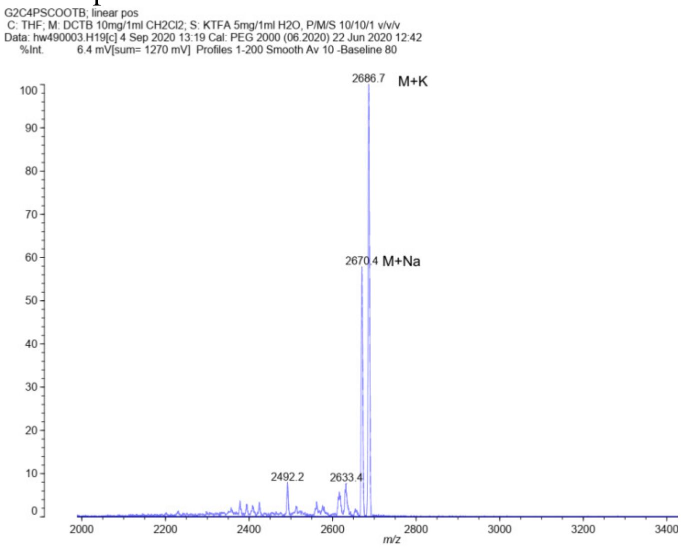

Spectra of the dendrimer **18** (**19**).

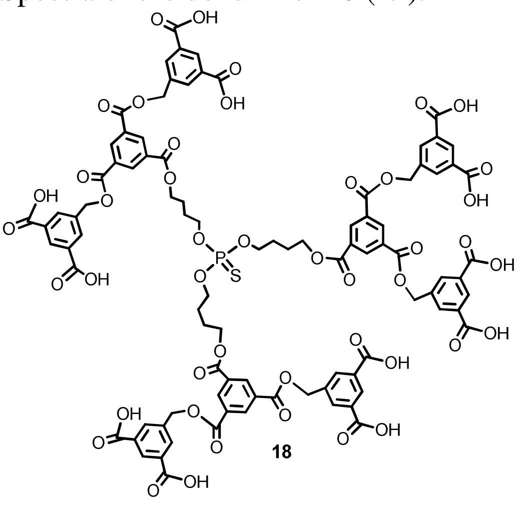

18

^{1}H NMR

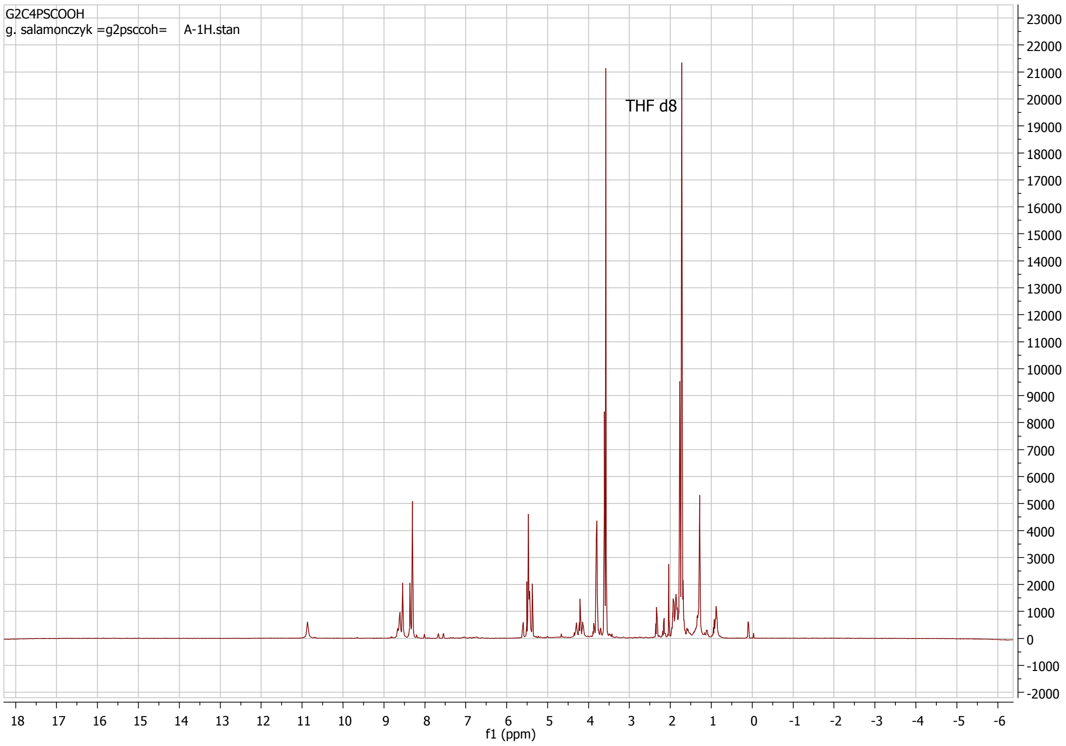

^{1}H {^{31}P} NMR

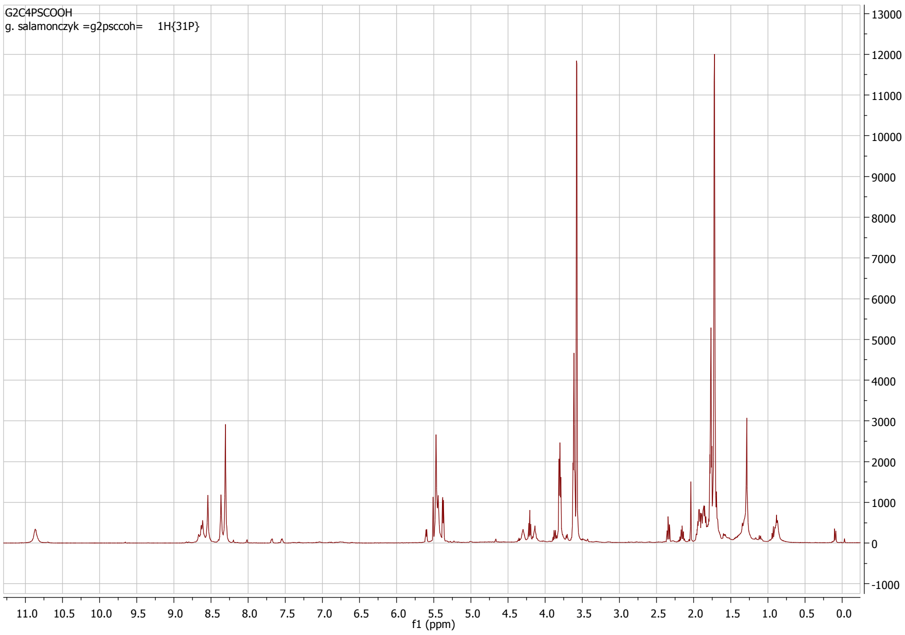

^{13}C NMR

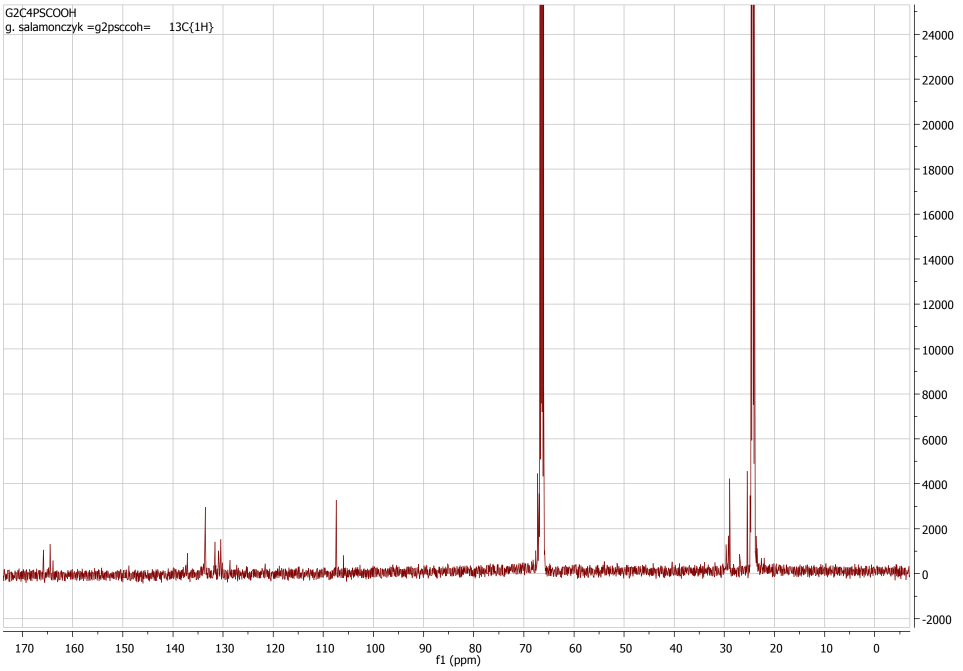

^{31}P NMR

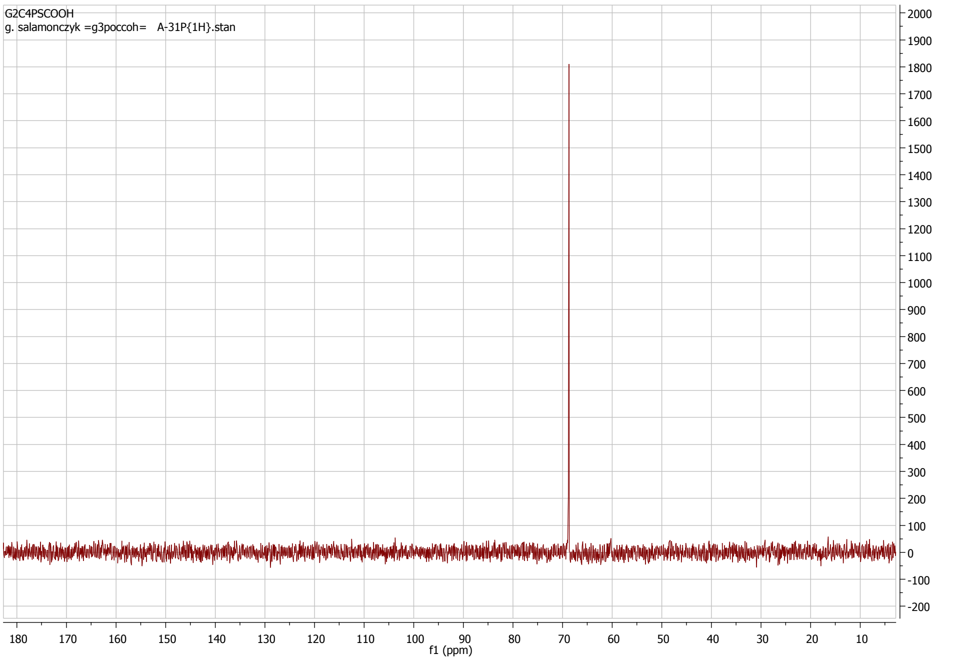

Spectra of the dendrimer **21**

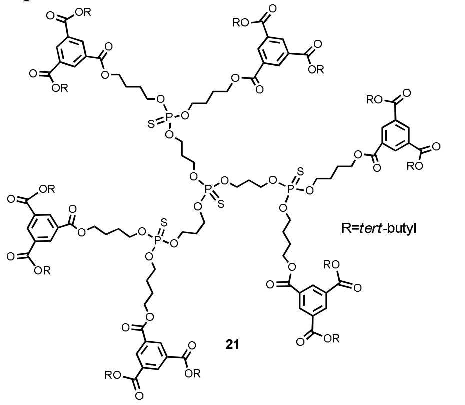

^{1}H NMR

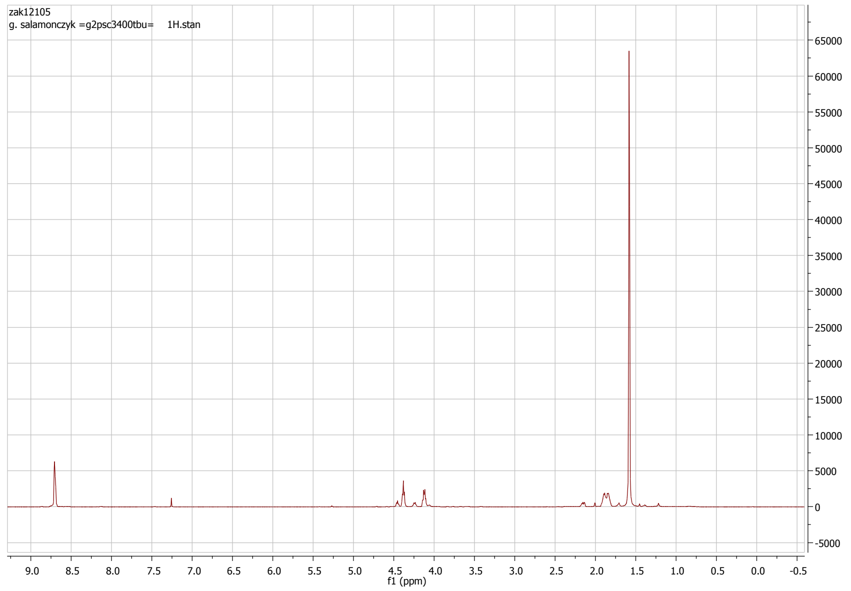

^{13}C NMR

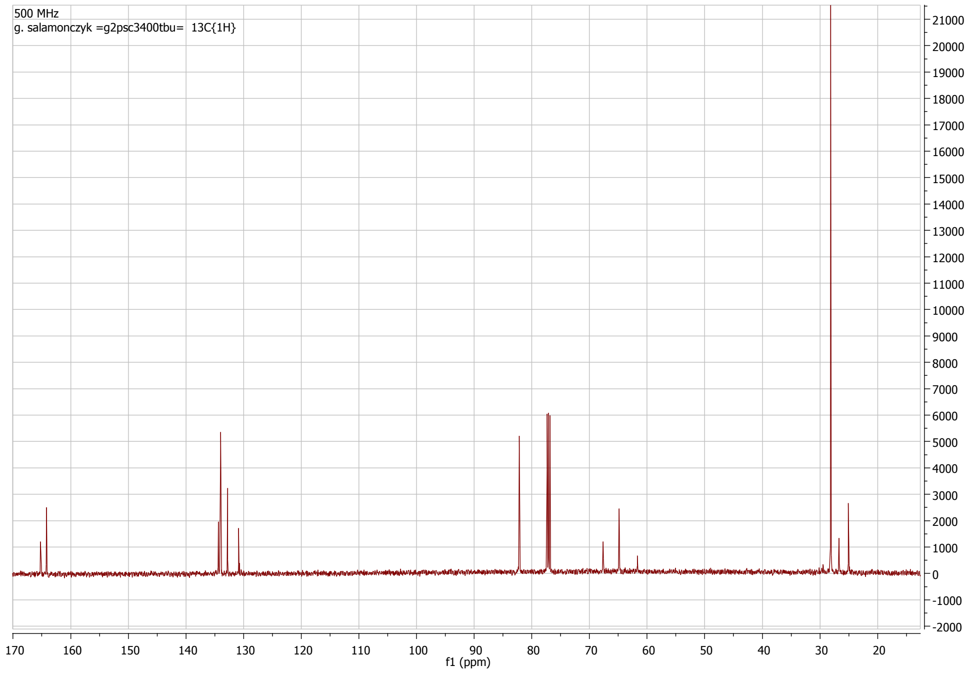

^{13}C NMR – DEPT 135

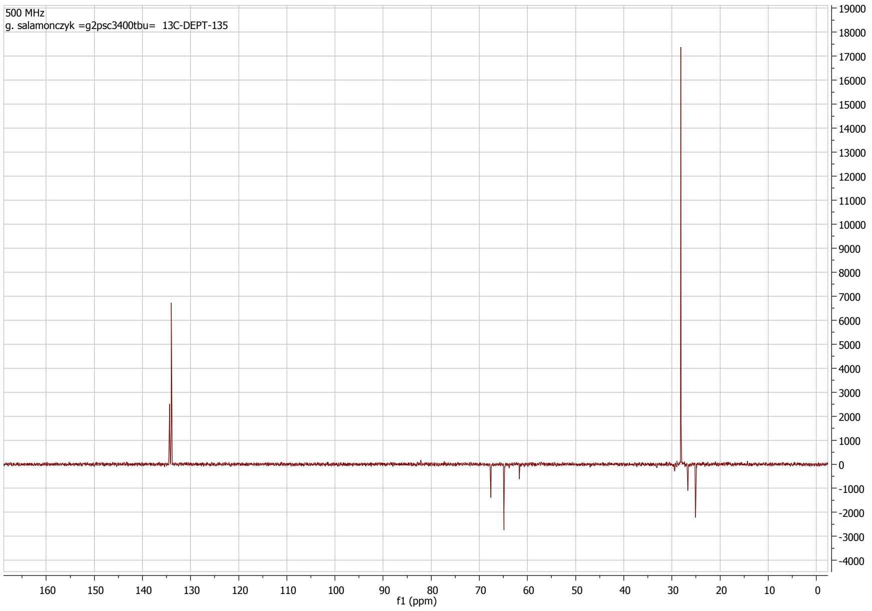

^{31}P NMR

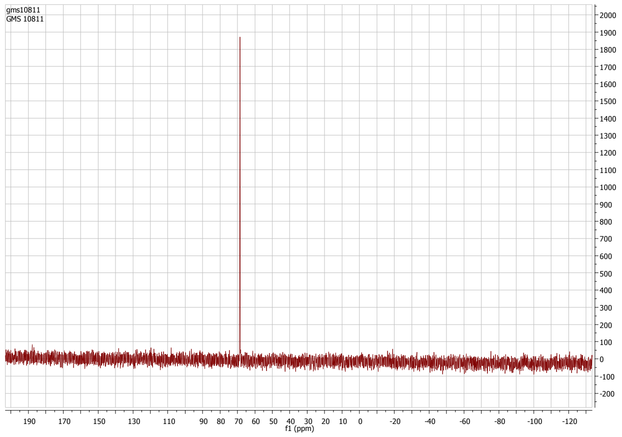

^{31}P NMR-expansion

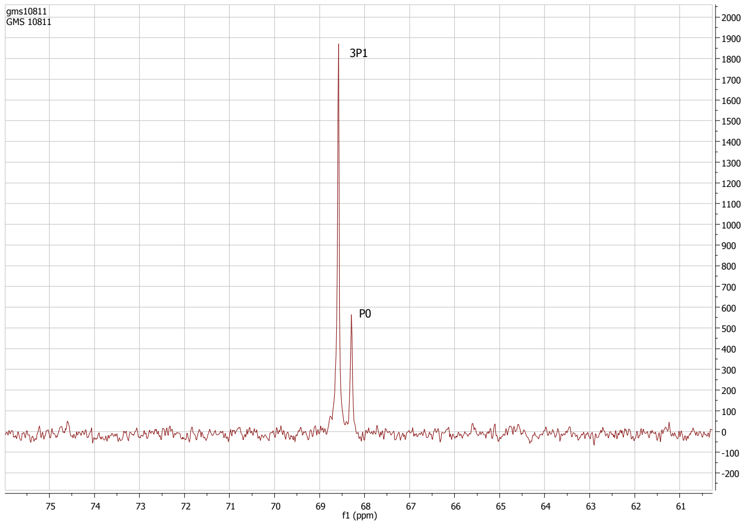

Spectra of the dendrimer **22** (**23**)

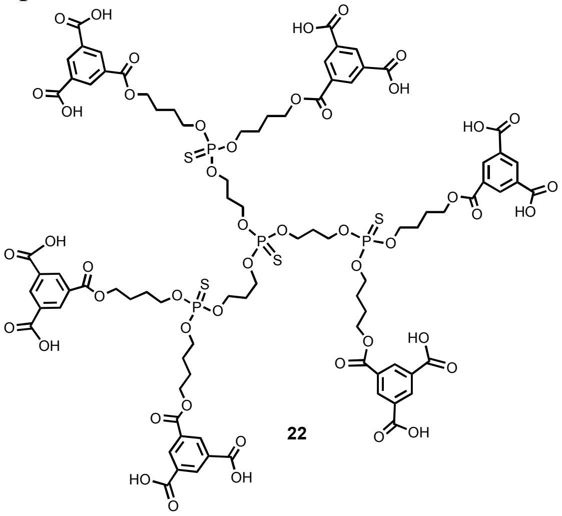

22

^{1}H NMR

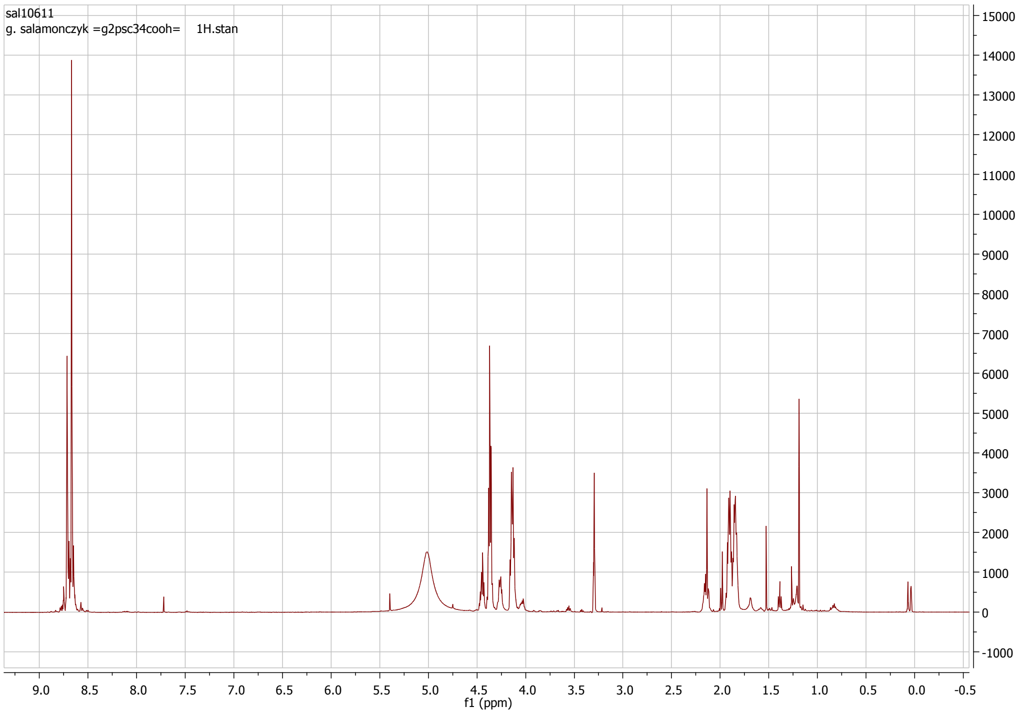

^{13}C NMR

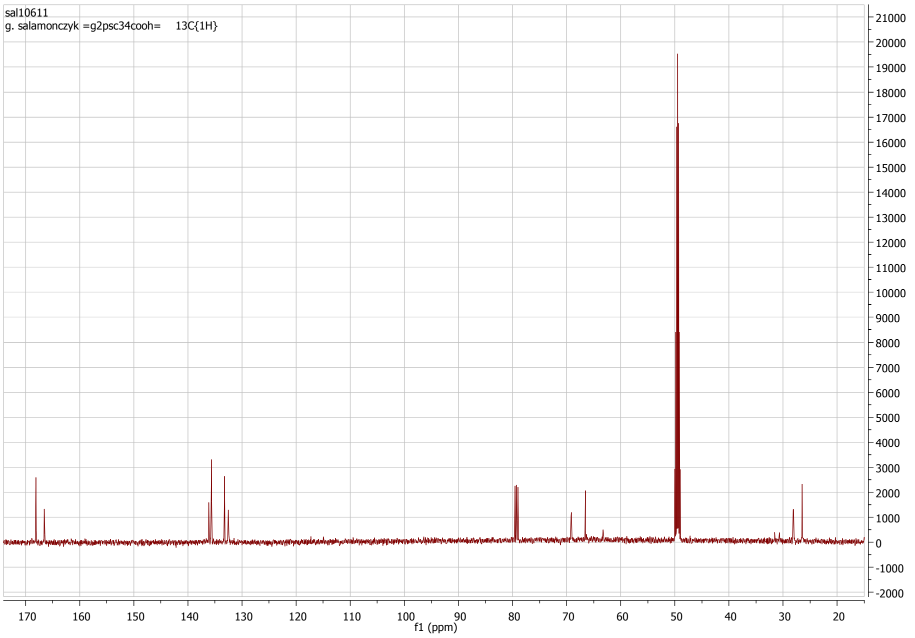

^{13}C NMR – DEPT 135

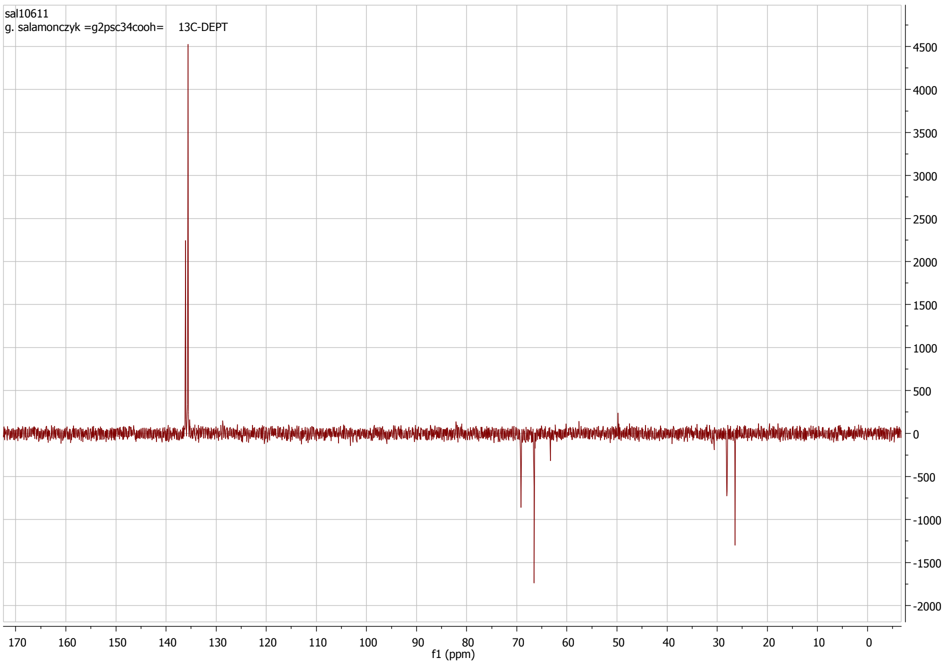

^{31}P NMR

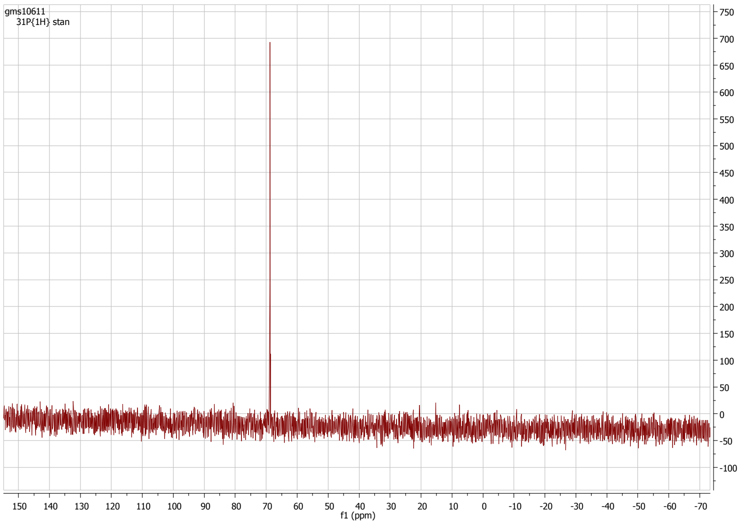

^{31}P NMR-expansion

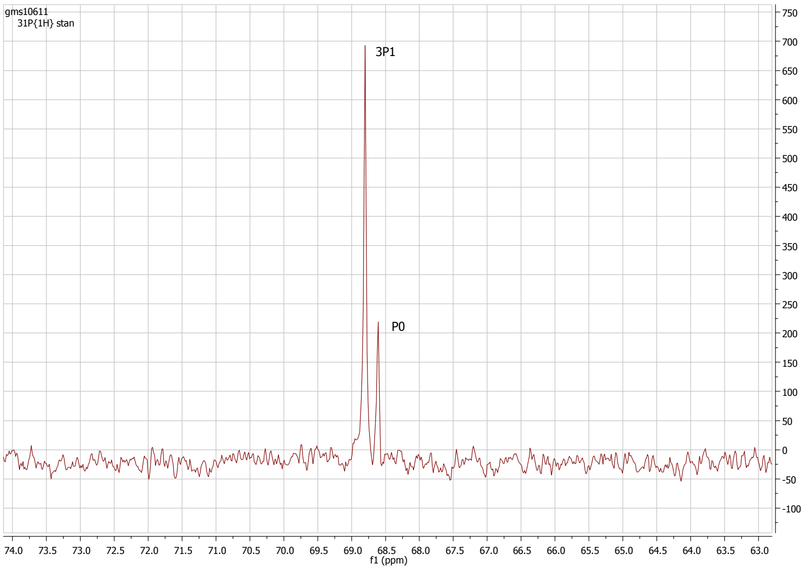

Supplement: Supplementary file 1 [file molecules-26-04754-s001.zip › molecules-1330229-supplementary.pdf]
